# Parental characteristics and offspring mental health and related outcomes: a systematic review of genetically informative literature

**DOI:** 10.1038/s41398-021-01300-2

**Published:** 2021-04-01

**Authors:** Eshim S. Jami, Anke R. Hammerschlag, Meike Bartels, Christel M. Middeldorp

**Affiliations:** 1 Department of Biological Psychology, Vrije Universiteit Amsterdam, Amsterdam, the Netherlands; 2 Department of Clinical, Educational and Health Psychology, Division of Psychology and Language Sciences, University College London, London, UK; 3 Amsterdam Public Health Research Institute, Amsterdam University Medical Centres, Amsterdam, the Netherlands; 4 Child Health Research Centre, University of Queensland, Brisbane, QLD Australia; 5 Child and Youth Mental Health Service, Children’s Health Queensland Hospital and Health Service, Brisbane, QLD Australia

**Keywords:** Human behaviour, Pathogenesis

## Abstract

Various parental characteristics, including psychiatric disorders and parenting behaviours, are associated with offspring mental health and related outcomes in observational studies. The application of genetically informative designs is crucial to disentangle the role of genetic and environmental factors (as well as gene–environment correlation) underlying these observations, as parents provide not only the rearing environment but also transmit 50% of their genes to their offspring. This article first provides an overview of behavioural genetics, matched-pair, and molecular genetics designs that can be applied to investigate parent–offspring associations, whilst modelling or accounting for genetic effects. We then present a systematic literature review of genetically informative studies investigating associations between parental characteristics and offspring mental health and related outcomes, published since 2014. The reviewed studies provide reliable evidence of genetic transmission of depression, criminal behaviour, educational attainment, and substance use. These results highlight that studies that do not use genetically informative designs are likely to misinterpret the mechanisms underlying these parent–offspring associations. After accounting for genetic effects, several parental characteristics, including parental psychiatric traits and parenting behaviours, were associated with offspring internalising problems, externalising problems, educational attainment, substance use, and personality through environmental pathways. Overall, genetically informative designs to study intergenerational transmission prove valuable for the understanding of individual differences in offspring mental health and related outcomes, and mechanisms of transmission within families.

## Introduction

**Fig. 1:**
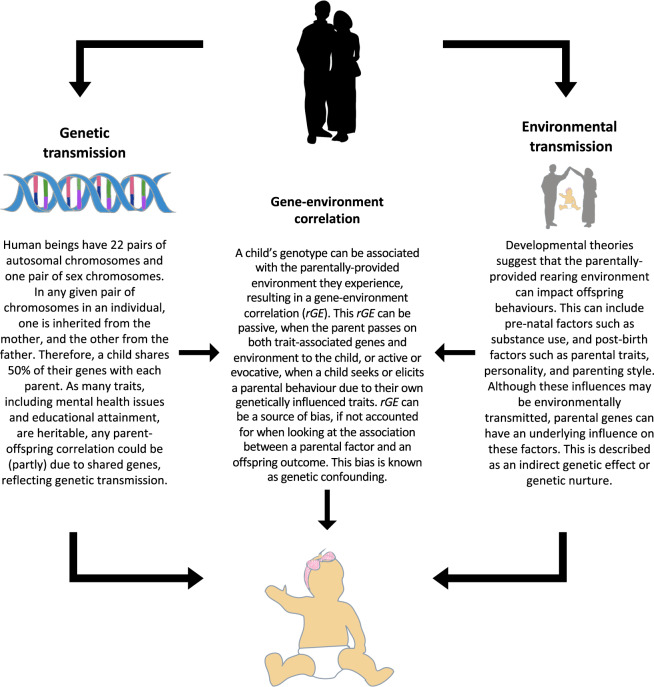
Mechanisms underlying parent-offspring associations. A figure describing potential mechanisms (genetic transmission, environmental transmission, and gene-environment correlation) underlying associations between parental characteristics and offspring outcomes.

Parents are considered a driving force in the development of their children and parental factors are associated with various mental health outcomes in offspring, including emotional and behavioural problems^1^. However, although observed associations between parental factors and offspring outcomes are often interpreted as direct environmental influences, in truth parents provide both the rearing environment and genes to their children. Thus, observed parent–offspring associations may be wholly or partially explained by genetic factors shared between the parent and child; i.e. in a gene–environment correlation (*rGE*), when exposure to specific environments depends on an individual’s genotype. The potential mechanisms (genetic transmission, environmental transmission and gene–environment correlation) underlying associations between parental characteristics and offspring outcomes are described in detail in Fig. 1. Designs that do not account for the role of genetic factors in parent–offspring correlations can lead to biased estimates and erroneous conclusions about the extent to which these associations are causal. Genetically informative designs that explicitly model or control for potential genetic effects are essential for improving our understanding of the true effect of the parentally provided environment on offspring mental health.

In genetic epidemiology, the classical twin design is generally used to decompose the contribution of genetic and environmental effects underlying human traits^2^. Twin-based research shows that most mental health and related traits are moderately heritable (under the influence of additive genetic effects), with additional variance explained by the unique environment (which is specific to each individual), and for some traits also the shared environment (environments that the twins have in common)^3^. However, classical twin studies say little about mechanisms of transmission within families where, in addition to genetic transmission, parental effects may be transmitted through both the shared environment via parentally provided rearing factors, and to a lesser extent, the unshared environment through specific parent–child interactions. Consequently, genetically informative designs that include both the parent and offspring generations are required to disentangle genetic and environmental effects underlying parent–offspring associations.

The present review aims to synthesise literature investigating the association between parental characteristics and offspring mental health and related outcomes in genetically informative designs. An earlier systematic review published in 2014 focused on the children-of-twins method^4^. However, several novel methodologies that investigate within-family transmission using innovative techniques have emerged in the past few years. Consequently, there is a gap in the literature for a broad systematic overview that incorporates all genetically informative designs that can be applied to study parent–offspring associations. Here, we focus on studies published from 2014 onwards, as these have not been covered by previous reviews. We first provide a brief overview of the types of genetically informative designs that can be employed to investigate parent–child associations. This is followed by a systematic review of studies investigating associations between parental characteristics and offspring mental health and related outcomes, including internalising behaviours (such as anxiety and depression), externalising behaviours (such as attention-deficit/hyperactivity disorder), educational attainment, substance use and personality.

## Genetically informative designs

**Fig. 2:**
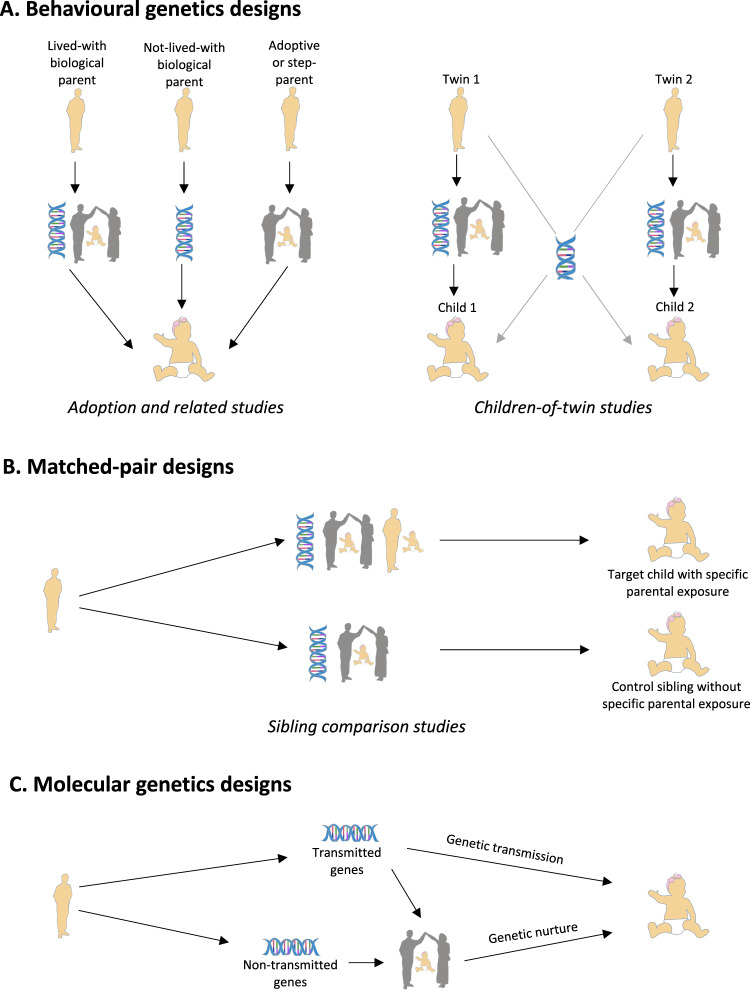
Schematic diagrams demonstrating the principles underlying commonly used genetically informative designs which separate genetic and environmental mechanisms of transmission in parent–offspring associations. **A** In adoption and related designs, knowledge of the type of relationship shared between parent and offspring is leveraged to gain insight into genetic and environmental factors underlying parent-to-offspring associations. Lived-with biological parents can influence offspring through both genetic and environmental transmission, as they provide both genes and the rearing environment. Not-lived-with biological parents who have no contact with the offspring provide only genes, indicating genetic transmission, whereas adoptive or step-parents provide only the rearing environment, indicating environmental transmission. In children-of-twins studies, children of identical (monozygotic) twins are as genetically similar to their aunt/uncle as they are to their parents (50% shared genes), whereas children of fraternal (dizygotic) twins share 25% of genes with their aunt/uncle. Higher monozygotic than dizygotic avuncular correlations (between uncle/aunt and niece/nephew, i.e. between Twin 1 and Child 2 or Twin 2 and Child 1) are likely due to a higher proportion of shared genes, suggesting genetic transmission, whereas higher parent–offspring than avuncular correlation suggests environmental transmission of a parental factor, above and beyond the effect of shared genetic or environmental effects. **B** In sibling comparison studies, the association between a specific parental factor and offspring outcome is studied in exposed versus unexposed offspring, as siblings are naturally matched for parentally provided genes and a rearing environment. Environmental transmission is indicated if the parent–offspring association is observed only in the exposed offspring. **C** In molecular genetics studies, the effect of shared parent–offspring (i.e. transmitted) genes on offspring outcome indicates the presence of genetic transmission. However, both transmitted genes and non-transmitted parental genes can also have an indirect (i.e. environmentally mediated) effect on offspring through parental traits that are genetically influenced; this is otherwise known as genetic nurture.

Designs that can be used to separate genetic and environmental mechanisms of transmission from parents to offspring broadly fall into the following three categories: behavioural genetics designs, matched-pair designs, and molecular genetics designs. In this section, we summarise the principles underlying these approaches (Fig. 2), describe specific methods in detail and discuss their application as well as advantages and disadvantages (Table 1).

**Table 1:** Overview of current designs that can be used to study mechanisms of transmission underlying associations between parental characteristics and offspring outcomes.

Design, reference	Genetic transmission	Environmental transmission	Gene–environment correlation	Advantages	Disadvantages
**Behavioural genetics designs**					
Adoption^5^	Association between a biological parent and their adopted-away offspring (shared genes only) indicates genetic transmission	Adoptee’s relative method: association between a parent and their adoptive offspring (rearing only) indicates environmental transmission Adoptee study method/siblings-reared-apart: higher correlation between a biological parent and their lived-with offspring (genes plus rearing) than their adopted- away offspring (genes only) indicates environmental transmission	Higher correlation between biological and living-together parents and offspring (genes plus rearing) than adoptive parents and offspring (rearing only) suggests passive *rGE* Trait correlation between biological parents and their adopted-away offspring (shared genes only) indicates genetic liability, and subsequent adoptee correlation with the environment provided by their adoptive parent suggests evocative *rGE*	- If adoption occurs at birth, passive *rGE* influences (on factors outside of the intrauterine environment) can be excluded as biological parents would have no rearing effect on the offspring	- Generalisability to the general population could be limited, as adoptees may have a higher risk of experiencing a suboptimal prenatal environment - Samples can be difficult to obtain and are usually small - Non-random process of adoption may introduce selection bias - Increase in open adoption (contact between biological and adoptive families) confounds the design
Assisted conception^7^	Higher correlation between a genetically related birth mother (e.g. homologous in vitro fertilisation or sperm donation) and her offspring (genes plus prenatal environment) than a genetically unrelated birth mother and her offspring (prenatal environment only) indicates genetic transmission	Association between a genetically unrelated birth mother (e.g. egg, oocyte or embryo donation, surrogacy) and her offspring (prenatal environment only) indicates environmental transmission	Not studied	- Effective for testing short and long-term effects of the prenatal environment	- Samples can be difficult to obtain and are usually small - Generalisability to the general population could be limited - Prenatal behaviours of mothers who use assisted conception may introduce selection bias - Samples are generally very heterogeneous - Inclusion of families with a within-family donation would bias the design - Design is not optimal for investigating gene–environment correlations
Triparental family (offspring-focused: multiple parental relationships of one offspring)^8^	Association between an offspring and their not-lived-with biological parent (genes only) indicates genetic transmission	Association between an offspring and their step-parent (rearing only) indicates environmental transmission	Higher offspring correlation with their lived-with biological parent (genes plus rearing) than with their step-parent (rearing only) suggests passive *rGE* Offspring correlation with their not-lived-with biological parent (shared genes only) indicates genetic liability, and subsequent offspring correlation with the environment provided by their step-parent suggests evocative *rGE*	- Representative of the general population as all types of parent–offspring relationships are included - Large sample sizes can be attained	- Contact with not-lived-with parents can upwardly bias estimate of genetic influences due to passive *rGE* - Databases with details of family structure are rare
Multiple parenting relationships (parent-focused; multiple offspring relationships of one parent)^9^	Association between a parent and their not-lived-with biological offspring (genes only) indicates genetic transmission	Association between a parent and their step-child (rearing only) indicates environmental transmission	Higher parental correlation with their lived-with biological children (genes plus rearing) than with their step-children (rearing only) suggests passive rGE	- Representative of the general population as all types of parent–offspring relationships are included - Large sample sizes can be attained	- Contact with not-lived-with parents can upwardly bias estimate of genetic influences due to passive *rGE* - Databases with details of family structure are rare - Cannot investigate evocative *rGE* as for each child in this design, information from only one parent is known
Children-of-twins^6^	Higher monozygotic–avuncular correlation (between MZ twin uncle/aunt and niece/nephew; 50% shared genes) than dizygotic–avuncular correlation (25% shared genes) indicates genetic transmission	Higher parent–child correlation (genes plus rearing) than monozygotic avuncular correlations (genes only) indicates environmental transmission	If a parental characteristic is largely estimated as heritable (under the effect of genes) in a parent-based twin sample but is under the influence of the shared environment in a child-based twin sample, this suggests passive *rGE* Estimation of a parental characteristic as heritable (under the influence of genest) in a child-based twin sample suggests evocative *rGE*	- Can determine if the familial correlation is due to genetic or environmental factors - Extended children-of-twins studies can incorporate siblings and other members of the pedigree and estimate additional parameters	- Samples can be difficult to obtain - Assumes that the size of the genetic contribution to variation in parent and offspring phenotype is the same - Assumes that the same genes influence the phenotype in both the parent and offspring generation
Extended twin (twins and their parents)^122^	Not studied, as genetic transmission is not estimated but fixed to 0.5 (50% of genes are passed on from parent to child) in the model*	The correlation between parental and offspring phenotype indicates cultural (i.e. environmental) transmission - this captures part of the shared environment effect that is explained by parent-to-child transmission	Covariance between the additive genetic effect and parental transmission suggests passive rGE	- Powerful for estimating shared environmental effects of a specific parental trait that arise due to cultural transmission or social homogamy - Design can be used to study the impact of other family relationships, including siblings - Design can be used to estimate twin-based heritability	- Cultural transmission can be easily underestimated if assumptions of the design are violated or the study is underpowered
**Matched-pairs designs**					
Sibling comparison^10^	Not studied, as the familial resemblance between full siblings could be due to genetic or environmental factors	Comparison of outcomes in children with a specific parental exposure and their unexposed full sibling who is otherwise naturally matched for familial (genetic and environmental) risk; higher outcome levels in exposed than unexposed siblings indicates environmental transmission	Not studied	- Generally excludes passive *rGE* as siblings typically share the same parentally provided environment - Can exclude evocative *rGE* within the design if certain that the parental exposure precedes offspring outcome	- Requires differential exposure between siblings, which can elicit selection bias - Cannot distinguish if the familial resemblance between siblings is due to genetic or environmental factors - Design is not optimal for investigating gene–environment correlations
Case–control^11^	Not studied, as cases and control parent–offspring pairs are matched on genetic risk	Parent-offspring pairs are manually matched on familial and genetic risk. Outcomes are compared between children with a specific parental exposure (cases) and unexposed children (controls); higher outcome levels in cases than controls indicates environmental transmission	Not studied	- Representative of the general population - If matched well, ensures no effect of confounding factors	- Matching is done by the researcher and is susceptible to errors - Resources required to find matched parent–offspring pairs - Cannot investigate genetic transmission or gene–environment correlation
**Molecular genetics designs**					
Within-family PGS:genetic sensitivity analysis^13^	The disappearance of an observed parent–offspring correlation after adjusting for offspring PGS for the predictor and outcome traits indicates genetic transmission	The remaining parent–offspring correlation, after adjusting for offspring PGS for the predictor and outcome traits, estimates environmental transmission	Reduction of parent–offspring correlation after adjusting for offspring PGS suggests passive *rGE*	- Can test whether parent–offspring associations are partly due to shared genes	- PGS capture only a small proportion of heritability and cannot index the effect of all shared genes - Requires well-powered GWAS summary statistics
Within-family PGS: genetic nurture^14,83^	Association between PGS based on transmitted parental genes and offspring outcome indicates genetic transmission	Transmitted/non-transmitted method: association between PGS based on non-transmitted parental genes and offspring outcome indicates genetic nurture Statistical control method: association between parental PGS and offspring outcome, after adjusting for offspring PGS to account for shared parent–child genetic effects indicates genetic nurture	Association between offspring PGS and parenting suggests passive *rGE* Association of offspring PGS with parenting, after adjusting for parental PGS suggests evocative *rGE*	- Can examine environmental transmission without parental phenotypic information	- Requires well-powered GWAS summary statistics - Datasets with parent–offspring genotyped duos or trios are rare
Maternal-effects genome-wide complex trait analysis (M-GCTA)^16^	Not studied*	The estimated effect of maternal or paternal genetic nurture: variance in offspring outcome that is explained by the effect of maternal *or* paternal genotype (after accounting for transmitted genetic effects)	Covariance between direct genetic effect and genetic nurturing effect suggests passive *rGE*	- Can estimate the overall impact of genetic nurture from mother or father - Representative of the general population - Design can be used to estimate SNP-based heritability	- Cannot model both maternal and paternal genetic nurture effects at the same time - Large sample sizes are required to estimate multiple variance components based on genetic data - Datasets with parent–offspring genotyped duos or trios are rare
Relatedness disequilibrium regression^87^	Not studied*	The overall estimated effect of parental genetic nurture: variance in offspring outcome that is explained by the effect of mid-parent genotype (after accounting for transmitted genetic effects)	Covariance between direct genetic effect and genetic nurturing effect suggests passive *rGE*	- Can estimate the overall impact of genetic nurture from both parents combined - Representative of the general population - Design can be used to estimate SNP-based heritability	- Assumes that maternal and paternal genetic effects are the same and of equal magnitude - Large sample sizes are required to estimate multiple variance components based on genetic data - Datasets with parent–offspring genotyped trios are rare
Trio-GCTA^18^	Not studied*	The Eestimated effect of maternal and paternal genetic nurture: variance in offspring outcome that is separately explained by the indirect effect of maternal *and* paternal genotype (after accounting for transmitted genetic effects)	Covariance between direct genetic effect and genetic nurturing effect suggests passive *rGE*	- Can estimate the individual impact of genetic nurture from both parents in the same model - Representative of the general population - Design can be used to estimate SNP-based heritability	- Large sample sizes are required to estimate multiple variance components based on genetic data - Datasets with parent–offspring genotyped duos or trios are rare

*rGE* gene–environment correlation, *PGS* polygenic scores, *SNP-based heritability* variance in a target trait that is explained by the additive genetic effect of common genetic variants known as single-nucleotide polymorphisms.

*These designs can be used to estimate twin or SNP-based heritability for offspring outcomes, i.e. the proportion of variance in a phenotype that can be explained by genetic variation in the population under study. This does not directly index genetic transmission, although it is implicitly known that children receive their genes from their parents.

### Behavioural genetics designs

Behavioural genetics designs leverage knowledge of relatedness among individuals within a family to make inferences about the contribution of genetic and environmental factors underlying parent–offspring associations. The adoption^5^ and children-of-twins^4,6^ designs (Fig. 2) are key tools used to distinguish the effects of genetic and environmental transmission. Associations between biological parents and their adopted-away offspring suggest genetic transmission as although these parents and offspring are genetically related, the parents do not raise the child and hence have no environmental influence. On the other hand, associations between adoptive parents and offspring suggest environmental transmission as these parents and offspring are genetically unrelated, and are only connected through the environment. In children-of-twins studies, children of monozygotic twins are as genetically similar to their twin aunt/uncle as they are to their twin parent, whereas children of dizygotic twins share less genetic similarity with their aunt/uncle. Higher monozygotic than dizygotic avuncular correlations (between uncle/aunt and niece/nephew) are likely due to the higher proportion of shared genes, suggesting genetic transmission, whereas higher parent–offspring than monozygotic or dizygotic avuncular correlation indicates environmental transmission through the shared parent–child environment. Another key characteristic of adoption and children-of-twins studies is that they can be used to investigate *rGE* (Table 1). This is particularly important as even within genetically informative designs, unmeasured *rGE* can inflate estimates of genetic or environmental effects. For instance, if an observed parent–offspring association is present in both biological and adoptive duos, but the correlation is higher in biological (shared genes plus rearing) than adoptive (rearing only) families, this indicates the contribution of both genetic and environmental effects; i.e. passive *rGE*. If unaccounted for, this *rGE*, reflected in increased similarity between biological parents and lived-with offspring, could potentially lead to an inflated estimation of genetic transmission in adoption studies.

Due to modern developments in assisted reproductive technology and the availability of large-scale population-based registers, novel pseudo-adoption designs have emerged that apply the same principles (see adoption and related designs in Fig. 1) to investigate genetic and environmental effects in non-adoption families. Within assisted conception^7^ studies, genetically related or genetically unrelated parents are analogous to the biological and adoptive parents in an adoption design, whereas in triparental family^8^ and multiple parenting relationships^9^ designs, the rearing effect of step-parents and genetic effect of not-lived-with biological parents are examined (Table 1).

### Matched-pair designs

Matched-pair designs strengthen the causal inference of an observed parent–offspring association by adjusting for all unmeasured genetic and environmental familial effects. In sibling comparison^10^ studies (Fig. 2), a sibling with no exposure to the parental candidate environment is included in the analysis as a control, as siblings are naturally matched for shared genes and the family environment. Environmental transmission is indicated if the parent–offspring association is observed only in the exposed offspring. Similarly, the case–control^11^ design includes matched parent–child control pairs who share the same proportion of genetic and environmental factors as the case parent–child pairs, but do not share the candidate exposure. As the matching is done by the researchers here, it is crucial that the process is thorough so that it can be reasonably argued that unmeasured confounders are unlikely to bias the results. Matched-pairs designs cannot be used to investigate *rGE*, as they do not directly measure genetic effects. However, sibling comparison studies generally rule out passive *rGE*, as the random distribution of parental alleles across offspring ensures that siblings are equally likely to receive genes associated with the exposure in the parent, and the outcome in the offspring. Evocative *rGE* can also be ruled out if exposure to the parental characteristic definitively precedes the offspring outcome.

### Molecular genetics designs

Recent advances in molecular genetics provide novel ways of investigating genetic and environment effects underlying parent–offspring associations by using genomic data. In molecular genetics studies, the effect of genetic variants transmitted from parent-to-offspring on offspring behaviour indicates the presence of genetic transmission. As described in Figs. 1 and 2, parental genes can also have an indirect effect on offspring, through parental traits that are environmentally mediated but genetically influenced; a process otherwise known as genetic nurture. One way to separate genetic transmission and genetic nurture effects underlying specific parent–offspring associations is the use of polygenic scores. Polygenic scores (PGS) represent an aggregate genetic liability for a trait, determined by the presence and effect sizes of alleles associated with the trait^12^. In within-family PGS genetic sensitivity analysis, offspring PGS for exposure and outcome traits are included as covariates in the regression analyses to explore whether the association between a parental exposure variable and offspring outcome is attenuated by the offspring’s PGS. If that is the case, genetic transmission explains part of the parent–offspring association^13^. Although adjusting for PGS does not entirely eliminate genetic transmission as current PGS capture only a small proportion of trait heritability, such sensitivity analyses can show whether shared genes partially account for an observed parent–offspring association. In within-family PGS genetic nurture analyses, PGS can additionally be used to estimate the environmental influence of parental alleles not passed on to the offspring^14,15^. If PGS based on non-transmitted parental alleles are associated with an offspring trait (transmitted/non-transmitted method in Table 1), the effect of these parental genes on offspring behaviour likely occurs via an environment pathway, i.e. genetic nurture. Similarly, if parental PGS are associated with an offspring trait, after adjusting for the child’s own PGS (statistical control method in Table 1), this also suggests a nurturing effect of parental genes beyond that which is due to transmitted genes (see statistical control method in Table 1). The overall contribution of genetic nurture to offspring traits can be estimated using maternal-effects genome complex trait analysis (M-GCTA)^16^, relatedness disequilibrium regression (RDR)^17^ or trio-GCTA^18^ (Table 1). Each of these methods uses genotyped data from unrelated parent–offspring pairs to estimate the variance in offspring behaviour that is explained by their own genotype (SNP-based heritability; heritability accounted for by differences in measured genetic variants known as single-nucleotide polymorphisms) and genetic nurture (parental additive genetic effects acting via genetically influenced parental behaviours).

It is important to note that as current genetic nurture designs only index parental effects that are captured by their common genetic variation, these designs capture only a part of the overall parent-to-child environmental transmission. Parental traits that are not under the influence of common genetic variation may also influence offspring outcomes. To test whether specific parental behaviours are responsible for observed genetic nurturing effects, the parental phenotype can be included as a covariate in within-family genetic nurture analyses, M-GCTA, RDR or trio-GCTA. If a genetic nurturing effect on offspring behaviour is attenuated with the inclusion of the parental phenotype to the model, the parental phenotype is shown to be involved in the manifestation of the genetic nurturing effect. As with behavioural genetics designs, molecular genetics designs can be used to investigate *rGE*, by estimating covariance between additive genetic effects and indirect genetic nurturing effects (Table 1).

## Methods

We searched for articles investigating associations between parental characteristics and offspring mental health and related outcomes. We defined related traits as those that have an established link to mental health in the literature. The Web of Science database was used to conduct a systematic search of studies published from 2014 to June 2020. The search terms consisted of study design variables (“children-of-twins” or “offspring of twins” or “adoption” or “assisted conception” or “sibling comparison” or “genetic nurture” or “non-transmitted” or “polygenic score”), parent variables (“parent” or “mother” or “father” or “maternal*” or “paternal*”), offspring variables (“offspring” or “child*”) and topic variables (“gene*” or “environment”). The search did not include predictor or outcome-specific search terms, so as not to limit the review to a particular set of traits. We restricted the search to scientific articles published in English. Through the results of the initial search, we identified additional designs that were relevant (Table 1), and ran separate follow-up searches for these study design variables (“extended twin” or “triparental” or “multiple parenting relationships design” or “matched pair” or “genome-wide complex trait analyses” or “relatedness disequilibrium regression”). Aside from the database searches, we scanned the references of papers for relevant studies and checked bioRxiv and medRxiv for relevant preprints.

After removing duplicates, the overall search yielded 2097 hits. Studies were included in the systematic review when the following criteria were met: the association between a parental characteristic and offspring behaviour was examined, a genetically informative design was used, and the phenotype in the offspring was a mental health or related trait. As current literature shows that most complex traits have a polygenic architecture, candidate gene studies were excluded from this review.

## Results

**Fig. 3:**
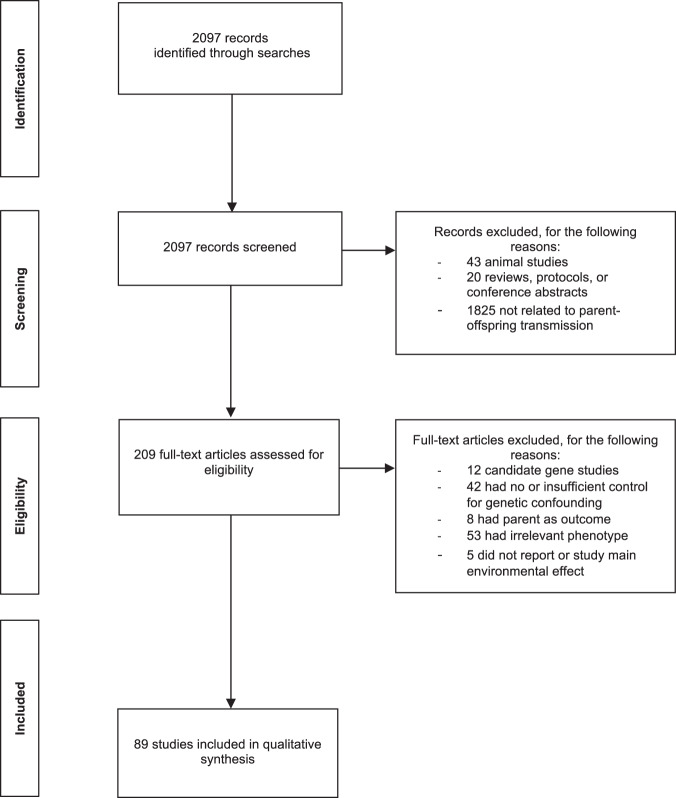
Flow chart of study selection. A description of the screening and assessment procedure, reporting the number of records excluded and reasons for exclusion at each stage.

After screening and assessment of search results (Fig. 3), we identified 89 articles for inclusion in this review. We present our synthesis of the literature by grouping the studies according to the offspring outcome in the following sections: internalising behaviours, externalising behaviours, educational attainment, substance use and personality. The number of studies and key findings for each outcome are summarised in Table 2. Details of all studies and their results are reported in Tables 3–7. Effect sizes showing the relative contribution of genetic and environmental factors in parent–offspring associations are included in the tables when studies provided standardised, well-interpretable statistics, i.e., odds ratios, percentage of variance explained or standardised betas.

**Table 2:** Summary of findings from the reviewed studies.

Trait(s)	Number of studies	Designs	Genetic overlap	Environmental transmission	Gene–environment correlation
**Offspring internalising behaviours**					
Parental internalising behaviours	19	11 adoption studies^19,20,25–28,30,32,36,41,57^ Five children-of-twins studies^19,21,22,29,123^ Three sibling comparison studies^24,31,34^ One multiple parenting relationships study^23^	There was evidence of genetic overlap between parental depression and offspring internalising symptoms, but not parental anxiety	There was evidence that parental anxiety and depression were associated with offspring internalising symptoms through environmental pathways	No evidence of evocative *rGE* was found, but child-to-parent effects were identified
Parenting	8	Four children-of-twins studies^37–40^ Two sibling comparison studies^42,65^ Two adoption studies^36,41^	There was no evidence of genetic overlap between parenting factors and offspring internalising behaviours	There was evidence that negative parenting behaviours were associated with more offspring internalising behaviours, and positive parenting was associated with fewer offspring internalising behaviours	No evidence of evocative *rGE* was found, but child-to-parent effects were identified
Genetic nurture	2	One M-GCTA study^43^ One RDR study^44^	Not studied	There was evidence of a genetic nurturing effect on offspring depressive (but not anxiety) symptoms	One study reported a negative *rGE* between genetic nurture and offspring depressive symptoms
Parental educational attainment	1	One children-of-twins and siblings study^45^	There was evidence of genetic overlap between parental educational attainment and offspring depressive symptoms	Parental educational attainment was not associated with offspring depression through an environmental pathway	Not studied
Parental substance use	1	One sibling comparison study^47^	Not studied	Maternal drinking during pregnancy was associated with emotional reactivity and somatic complaints, but associations with anxiety and depressive symptoms were confounded	Not studied
**Offspring externalising symptoms**					
Parental externalising behaviours	9	Seven adoption studies^48–54^ One multiple parenting relationships study^55^ One triparental study^8^	There was evidence of genetic transmission of criminal behaviours; evidence for other externalising symptoms was ambiguous, although better-powered studies tended to find supportive evidence	There was evidence that parent and offspring criminal behaviours were associated with environmental pathways	Not studied
Parental internalising behaviours	11	Six adoption studies^19,20,25,41,51,57^ Three children-of-twins studies^19,22,56^ Three sibling comparison studies^21,24,31^	There was evidence of genetic overlap between parental depression and offspring externalising symptoms	There was evidence that parental depression was associated with offspring internalising symptoms through environmental pathways	One study reported evocative *rGE* effects on the association between parental depression and offspring externalising symptoms
Parenting	14	11 adoption studies^41,48–53,57,60,61,64^ Two sibling comparison studies^62,65^ One children-of-twins study^59^	There was some evidence of genetic overlap between parenting factors and offspring externalising behaviours	There was evidence that negative parenting behaviours were associated with more offspring externalising behaviours, whereas positive parenting was associated with fewer offspring externalising behaviours	There was some evidence of evocative *rGE* and other child-to-parent effects
Parental substance use	10	Seven sibling comparison studies^47,67,70–74^ Three adoption studies^51,64,66^ One children-of-twins study^67^	There was evidence of genetic overlap between parental drug abuse and smoking and offspring externalising behaviours	There was mixed evidence for an environmental association between parental substance use and offspring externalising behaviours	Not studied
Parental education	3	One within-family PGS genetic sensitivity study^13^ One within-family PGS genetic nurture study^75^ One children-of-twins study^45^	There was evidence of genetic overlap between parental educational attainment and offspring externalising symptoms	There was some evidence of environmental associations between maternal education and offspring attention-deficit hyperactivity disorder (ADHD)	Not studied
Genetic nurture	1	One within-family PGS study^75^	Not studied	No genetic nurturing effect on offspring ADHD was observed	Not studied
**Offspring educational attainment**					
Parental educational attainment	9	Four adoption studies^80,81,90,124^ Three within-family PGS genetic sensitivity studies^13,82,84^ One extended twin study^79^ One children-of-twins and siblings study^45^	There was substantial evidence of genetic overlap between parental and offspring educational attainment	There was evidence that parent and offspring educational attainment were associated through environmental pathways	One study reported passive *rGE* effects underlying the association between parent and offspring educational attainment
Genetic nurture	12	11 within-family PGS studies^14,15,75,78,82–86,88,89^ One RDR study^17^	Not studied	There was evidence of a genetic nurturing effect on offspring educational attainment	There was evidence of passive *rGE* on offspring educational attainment
Maternal smoking during pregnancy	2	Two sibling comparison studies^67,71^ One children-of-twins study^67^	There was evidence of genetic overlap between maternal smoking during pregnancy and offspring cognition	There was little evidence of environmental associations between maternal smoking during pregnancy and offspring cognition	Not studied
**Offspring substance use**					
Parental substance use behaviours	15	Four children-of-twin studies^67,94,96,100^ Two adoption studies^95,101^ Two sibling comparison studies^67,105^ Two triparental studies^8,98^ Two multiple parenting relationships studies^9,99^ Two extended-family designs^42,97^ One within-family PGS genetic sensitivity analysis study^100^ One extended twin study^103^ One matched-pair case–control study^11^	There was evidence of genetic overlap between parental and offspring substance use behaviours	There was evidence of environmental associations between parental and offspring substance use behaviours	One study reported passive *rGE* underlying the association between parent and offspring substance use behaviours
Parenting	4	Three adoption studies^104,107,108^ One sibling comparison study^109^	Not studied	There was evidence of protective effects of several parental factors on offspring substance use behaviours	Not studied
**Offspring personality**					
Parental characteristics	6	Three adoption studies^61,110,111^ One sibling comparison study^71^ One children-of-twins study^29^ One extended twin study^112^	There was some evidence of genetic overlap between parental characteristics and offspring personality	There was some evidence of environmental associations between parental and offspring personality	There was evidence of evocative *rGE* underlying associations between parenting behaviours and offspring social behaviours

*rGE* gene–environment correlation, *M-GCTA* maternal-effects genome-wide complex traits analysis, *RDR* relatedness disequilibrium regression, *PGS* polygenic scores.

**Table 3:** Detailed characteristics of studies investigating offspring internalising behaviours (*N* = 30).

Offspring internalising behaviours								
Study	Design	Sample	Parental attribute (predictor)	Child attribute (outcome)	Control variables	Genetic overlap	Environmental transmission	G–E interplay
Brooker et al.^27^	Adoption	*EGDS* 361 families Offspring age: 18–27 months	*Adoptive & birth parent anxiety* *:* self-report, BAI	*Internalising problems:* maternal and paternal report, composite score, CBCL		No, birth parent anxiety did not predict offspring internalising problems	Yes, adoptive parent anxiety predicted offspring internalising problems (*β* = 0.25)	G × E: high birth parent anxiety × greater attention control × low adoptive parent anxiety: fewer internalising problems
Brooker et al.^28^	Adoption	*EGDS* 349 families Age: 9–27 months	*Adoptive parent anxiety:* self-report, BAI *Birth parent negative affect:* self-report, ATQ	*Negative affect:* observation and adoptive-parent report, composite score, ICQ and TBAQ	Prenatal risk and obstetric complications, adoption openness	No, birth parent negative affect did not predict offspring negative effect (effect size not clear)	Yes, adoptive parent anxiety predicted offspring negative effect (effect size not clear)	No evidence of evocative *rGE*, but child-to-parent effects found
Marceau et al.^41^	Adoption	*EGDS* 361 families Age: 9 months 6 years	*Over-reactive parenting:* self-report, PS *Birth mother risk:* self-report, composite score, substance use, depression (BDI) & anxiety (BAI)	*Internalising behaviours:* parent report, CBCL	Adoption openness	No, birth mother risk did not predict offspring internalising behaviours (effect size not clear)	Yes, paternal (but not maternal) over-reactive parenting predicted offspring internalising behaviours (effect size not clear)	
McAdams et al.^19^	Adoption, Children-of- twins	*Adoption: EGDS* 361 families Age: 4.5–7 years *CoT: TOSS* 287 monozygotic (MZ) & 489 dizygotic (DZ) twin families Age: 11–22 years	*Adoptive & parent depression:* self-report, BDI *Depressive symptoms (CoT):* self-report, CES-D	*Internalising problems (adoption sample):* parent report, CBCL *Internalising problems (CoT sample):* mother, father and self-report, CBCL	Adoption sample: obstetric complications, adoption openness CoT sample: twin sex, age	Adoption: birth mother depressive symptoms predicted internalising problems at age 7 (*β* = 0.15), but not age 4.5 or age 6 CoT: no shared genetic effects between parental depression and offspring internalising problems	Adoption: no, adoptive parent depression did not predict subsequent offspring internalising problems CoT: after accounting for genetic relatedness, parental depression was associated with offspring internalising problems (effect size not clear)	No evidence of evocative *rGE*, but child-to-parent effects found
Eley et al.^29^	Children-of-twins	*TOSS* 387 MZ, 489 DZ families Age: 11–22 years	*Anxious personality:* self-report, KSP	*Anxiety:* mother, father and self-report, CBCL	Twin sex, age	No shared genetic effects between parental anxious personality and offspring anxiety	Yes, after accounting for genetic relatedness, parental anxiety was associated with offspring anxiety symptoms (effect size not clear)	
Roos et al.^57^	Adoption	*EGDS* 293 families Age: 6–7 years	*Adoptive & birth mother internalising symptoms:* self-report, composite score, BAI and BDI *Adoptive mother uninvolved parenting:* self-report, APQ *Adoptive & birth mother processing speed:* Stroop colour-word naming task	*Internalising-only problems:* parent report, CBCL *Co-occurring internalising and externalising problems:* parent report, CBCL	Child sex, child age, adoption openness, obstetric complications	Birth mother internalising symptoms and processing speed did not predict internalising-only symptoms, but processing speed was associated with co-occurring symptoms (OR = 1.88)	Adoptive parent internalising symptoms predicted internalising-only symptoms (OR = 1.17), but not co-occurring symptoms, uninvolved parenting predicted co-occurring symptoms (OR = 7.91), but not internalising-only symptoms and adoptive parent processing speed and offspring outcomes were unrelated	G × E: adoptive mother high internalising symptoms × inherited risk of slow processing speed: co-occurring symptoms
Grabow et al.^20^	Adoption	*EPoCH* 541 adoptive mother–child dyads, 126 biological mother–child dyads Age: 7 years	*Maternal trauma frequency:* repeated self-report, mean score, NLES *Adoptive & birth mother depressive symptoms:* self-report, BDI	*Internalising behaviours:* parent report, CBCL	EPoCH: Timing of maternal trauma, socioeconomic status (SES), sex EGDS: Perinatal risk, adoption openness, SES, sex	Yes, birth mother depression predicted adopted-away offspring internalising behaviours (*β* = 0.16)	Adopted mother depression predicted offspring internalising behaviours (*β* = 0.15), and mediated the relationship between maternal trauma and offspring internalising behaviours	
Gjerde et al.^24^	Sibling comparison	*MoBa* 17,830 siblings, 11,599 families Age: 6 months to 5 years	*Maternal depression:* self-report, SCL	*Internalising problems:* maternal report, CBCL	Maternal parity, maternal education, child age and child sex	Not studied	Children exposed to concurrent maternal depression had more internalising symptoms than their unexposed siblings, but perinatal maternal symptoms had no effect	
Bekkhus et al.^34^	Sibling comparison	*MoBa* 21,980 families with at least two siblings Age: 6 months to 3 years	*Maternal anxiety during pregnancy:* self-report, SCL (short version)	*Infant difficulties:* maternal report, ICQ *Emotional difficulties:* maternal report, CBCL	Maternal substance use during pregnancy, post-birth anxiety, partner disharmony, somatic disease, marital status, education, age, parity, child gestational age, birth complications, sex, birthweight	Not studied	No difference in infant difficulties or emotional difficulties between exposed and unexposed siblings	
Bridgett et al.^36^	Adoption	*EGDS* *361 families* *Age: 4.5–6 years*	*Harsh negative parenting:* observation *Biological parent self-regulation:* Go/no Go task computerised task	*Self-regulation:* parent report (Children’s Behaviour Questionnaire) and Go/no Go computerised task	Obstetric and neonatal complications, adoption openness, child anger (parent report), gender	Yes, birth mother self-regulation predicted adopted-away offspring’s self-regulation (*β* = 0.23)	Yes, adoptive parent harsh parenting predicted poor offspring self-regulation (*β* = −0.22 to −0.25)	No evocative *rGE*, but child-to-parent effects of child anger found
Hannigan et al.^21^	Multiple children-of-twins and siblings	*MoBa* 22,195 mothers, 25,299 children Age: 18–60 months	*Maternal depressive symptoms:* self-report, SCL	*Internalising problems:* maternal report, CBCL	Prenatal depression: adjusted for concurrent depression	Yes, there were shared genetic effects between maternal depression and offspring internalising problems effect size not clear)	Yes, after accounting for genetic relatedness and prenatal depression, concurrent maternal depression was associated with offspring internalising problems (effect size not clear)	
Liskola et al.^26^	Adoption	*FAS* 548 international adopted children Age: 9–12 years	*Depressive symptoms:* self-report, GHQ	*Depressive symptoms:* self-report, CDI	Child age, gender, age at adoption, type of placement before adoption, the continent of birth, adoptive family SES	Not studied	Adoptive paternal (but not maternal) depressive symptoms were associated with offspring depressive symptoms	
Kendler et al.^23^	Multiple parenting relationships design	*Snr* 2,041,816 intact, 14,104 adoptive, 115,501 not-lived-with father, 57,826 stepfather, 29,205 triparental families Age: 26–56 years	*Major depression:* diagnosis, hospital discharge and outpatient care registers	*Major depression:* diagnosis, hospital discharge and outpatient care registers	None	Yes, MD status of not-lived-with biological parents was associated with offspring MD (*r* = 0.08)	Yes, MD status of adoptive or step-parents was associated with offspring MD (*r* = 0.08)	No G × E interaction found
Ahmadzadeh et al.^30^	Adoption	*EGDS* 305 families Age: 6–8 years	*Adoptive parent anxiety:* self-report, ST-AIA *Birth parents’ internalising problems:* mother & father self-report, composite score, CIDI and FH-RDC	*Anxiety:* maternal and paternal report, CBCL	The weighted risk score of obstetric complications, adoption openness, child sex	No, birth parents’ internalising problems did not predict adopted-away offspring anxiety	Adoptive paternal anxiety (but not maternal) predicted offspring anxiety (*β* = 0.10)	No evocative *rGE*, but child-to-mother effects found
Gjerde et al.^22^	Multiple children-of-twins and siblings	*MoBa* 22,316 mothers and 35,589 offspring Age: 1.5–5 years	*Concurrent maternal depression symptoms:* self-report, SCL	*Emotional problems:* maternal report, CBCL	Child sex, maternal age	Yes, there were shared genetic effects between maternal depression and offspring emotional problems (*R*^2^ = 21.1–28.5%)	Yes, after accounting for genetic relatedness, maternal depression was associated with offspring internalising problems (*R*^2^ = 0.3–2.2%)	
Hails et al.^25^	Adoption	*EGDS* 561 families Age: 9 months to 6 years	*Adoptive parent depression:* self-report, BDI-IIBirth *mother internalising symptoms:* self-report, CIDI	*Internalising symptoms:* parent and teacher report, CBCL and (TRF	Adoption openness, prenatal risk and obstetric complications, infant negative emotionality	No, birth mother internalising symptoms did not predict offspring internalising symptoms	Adoptive paternal (but not maternal) depression predicted parent-reported (but not teacher-reported) offspring internalising symptoms (*β* = 0.21)	
Field et al.^32^	Adoption	*EGDS* 561 families Age: 18 months to 4.5 years	*Adoptive and birth parent anxiety:* self-report, composite score of two measurements, BAI	*Anxiety symptoms:* parent report, an average of the maternal and paternal report, CBCL		No, birth parent anxiety did not predict offspring anxiety symptoms	Adoptive maternal and paternal anxiety equally predicted both offspring anxiety symptoms and change in anxiety symptoms (effect size not clear)	No evidence of evocative *rGE* found
Gjerde et al.^31^	Sibling comparison	*MoBa* 11,553 mothers and 17,724 children Age: 1.5–5 years	*Maternal anxiety*: self-report, SCL	*Child internalising problems:* maternal report, CBCL	Child age, child sex, maternal depressive symptoms, parity, education	Not studied	Children exposed to concurrent maternal anxiety had more internalising symptoms than their unexposed siblings, but perinatal maternal symptoms had no effect	
O’Reilly et al.^123^	Children of siblings	*Snr* 2,762,883 unique offspring Age: 12 and over	*Suicidal behaviour:* suicide attempt or death by suicide, National Patient Register and Cause of Death register, prior to offspring age 18	*Suicidal behaviour:* suicide attempt or death by suicide, National Patient Register and Cause of Death register	Offspring: parity. Parental: age at birth, educational attainment, Swedish by birth, mental illness, criminal convictions	Yes, there were shared genetic effects between parental and offspring suicidal behaviour (effect size not clear)	Yes, after accounting for genetic relatedness, parental suicidal behaviour was associated with offspring suicidal behaviour effect size not clear)	
Horwitz et al.^37^	Extended children-of-twins	*TOSS, TCHAD* 858 twin families, 690 twin families Age: 11–22 years, 16–17 years	*Parental criticism:* self-report, EES	*Somatic symptoms:* parent and self-report, composite score, CBCL	Age, sex, age difference for the cousin offspring in TOSS	No shared genetic effects between parental criticism and offspring somatic symptoms	Yes, after accounting for genetic relatedness, parental criticism was associated with offspring somatic symptoms (effect size not clear)	No evidence of passive or evocative *rGE* found
Guimond et al.^65^	Sibling comparison	*QNTS* 164 twin pairs Age: 13–14 years	*Perceived maternal support and negativity:* child report, NRI	*Depressive symptoms:* self-report, CDI	Genetically-controlled analyses using MZ twin-difference score	Not studied	No, perceived maternal support and negativity were not associated with offspring depressive symptoms	No evidence of evocative *rGE*, but child-to-parent effects found
McAdams et al.^38^	Children-of-twins	*TOSS* 387 MZ, 489 DZ families Age: 11–22 years	*Expressed affection and closeness with child:* self-report	*Self-worth:* self-report, HPCS	Twin sex and age	No shared genetic effects between expressed affection or closeness with child and offspring self-worth	Yes, after accounting for genetic relatedness, expressed affection and closeness with the child were associated with offspring self-worth (effect size not clear)	
Hannigan et al.^39^	Children-of-twins	*TOSS* 909 twin pairs Age: 11–22 years	*Relationship quality with offspring:* maternal and paternal report, P-CAS, EAS and P-CRQ	*Internalising problems:* self-report, CBCL		No shared genetic effects between parental relationship quality with offspring, and offspring internalising problems	Yes, after accounting for genetic relatedness, parental relationship quality with offspring was associated with offspring internalising problems (effect size not clear)	
Ahmadzadeh et al.^40^	Extended children-of-twins	*TOSS, TCHAD* 876 twin families, 1030 twin families Age: 11–22 years	*Parental criticism:* self-report, EES	*Internalising symptoms:* parent and self-report, composite score, CBCL and YSR	Child age, sex	No shared genetic effects between parental criticism and offspring internalising symptoms	Yes, after accounting for genetic relatedness, parental criticism was associated with offspring internalising symptoms (effect size not clear)	
Kendler et al.^42^	Sibling comparison	*Snr* 666 full sibships and 2596 half-sibships of high-risk (MDD diagnosis) biological parents Age: 15 and over	*Adoptive parenting:* protective effect of high-quality rearing environment	*Major depression:* diagnosis, hospital discharge, outpatient care registers, primary care registry	Parental age at birth, high-risk status of the other parent of a half-sibling, child sex	Not studied	Children exposed to adoptive parenting had a lower risk of MDD than their unexposed siblings, this protective effect disappeared when the adoptive family was disrupted or if there was a high-risk adoptive parent	
Jami et al.^43^	M-GCTA, children-of-twins and siblings	*MoBa* M-GCTA: 3801 parent–offspring trios, extended CoT: 10,688 children Age: 8 years	*Genetic nurture*: M-GCTA, maternal and paternal genotypes *Shared maternal or paternal environment*: children-of-twins and siblings	*Anxiety symptoms:* maternal report, SCARED *Depressive symptoms:* maternal report, SMFQ	Sex, genotyping batch, first ten principal components	Not studied	After accounting for shared genetic effects, maternal or paternal genes did not explain significant variance in offspring depression or anxiety symptoms, and there were no shared maternal or paternal environment effects	No evidence of *rGE* found
Cheesman et al.^44^	Relatedness disequilibrium regression (RDR), children-of-twins and siblings	*MoBa* RDR: 11,598 parent–offspring trios, extended CoT: 26,086 pairs of relatives Age: 8 years	*Genetic nurture:* RDR, mid-parent genotype *Maternal emotional symptoms:* self-report, common factor score of 5 measurements, SCL-8 *Shared parental environment:* children-of-twins and siblings	*Anxiety symptoms:* maternal report, SCARED *Depressive symptoms:* maternal report, SMFQ	Child sex. RDR: ten principal components and genotyping batch	Not studied	After accounting for shared genetic effects, parental genes explained significant variance in offspring depression (but not anxiety) symptoms, this effect was partly mediated by maternal emotional symptoms Shared parental environmental effect was observed for offspring depression (but not anxiety) symptoms	Negative *rGE* between genetic nurture and offspring depressive symptoms
Lund et al.^47^	Sibling comparison	*MoBa* 14,639 mothers, 25,744 children Age: 1.5–5 years	*Maternal alcohol consumption during pregnancy:* self-report, AUDIT-C	*Emotional problems:* maternal report, CBCL *Emotional reactivity* *Anxious/depressed* *Somatic complaints*	Parity, unplanned pregnancy, daily smoking, pre-pregnancy abstinence from alcohol	Not studied	Exposed children were more emotionally reactive and had more somatic complaints, but did not have more anxious depressive symptoms, than their unexposed siblings	
Torvik et al.^45^	Children-of-twins and siblings	*MoBa* 34,958 children Age: 8 years	*Educational attainment* (EA*):* self-report, highest level completed	*Depression symptoms:* maternal report, SMFQ		Yes, there were shared genetic effects between parental EA and offspring depression symptoms (effect size not clear)	No, after accounting for genetic relatedness, parental EA was not associated with offspring depression	

*G–E* gene–environment, *G* *×* *E* gene–environment interaction, *rGE* gene–environment correlation.

Design = *M-GCTA* maternal-effects genome-wide complex traits analysis.

Samples = *EGDS* Early Growth and Development Study, *EPoCH* Early Parenting of Children study, *FAS* Finnish Adoption Study, *MoBa* Norwegian Mother Father and Child Study, *QNTC* Quebec Newborn Twin Study, *Snr* Swedish national registers, *TCHAD* Twin Study of Child and Adolescent Development, *TOSS* Twin Offspring Study of Sweden.

Measures = *APQ* Alabama Parenting Questionnaire, *ATQ* Adult Temperament Questionnaire, *AUDIT-C* Alcohol Use Disorder Identification Test-Consumption, *BAI* Beck Anxiety Inventory, *BDI* Beck Depression Inventory, *CBCL* Child Behaviour Checklist, *CDI* Children’s Depression Inventory, *CES-D* Centre for Epidemiological Studies Depression Scale, *CIDI* Composite International Diagnostic Instrument, *EAS* Expression of Affection Scale, *EES* Expression Emotion Scale, *FH-RDC* Family History-Research Diagnostic Criteria, *GHQ* General Health Questionnaire, *HPCS* Harter Perceived Competence Scale, *ICQ* Infant Characteristics Questionnaire, *KSP* Karolinska Scales of Personality, *NLES* Negative Life Events Scale, *NRI* Network of Relationships Inventory, *P-CAS* Parent–Child Agreement Scale, *P-CRQ* Parent–Child Relationship Questionnaire, *PS* the Parenting Scale*, SCARED* Screen for Child Anxiety Related Disorders, *SCL* Symptoms Checklist, *SMFQ* Short Mood and Feelings Questionnaire, *S-TAIA* State-Trait Anxiety Inventory for Adults, *TBAQ* Toddler Behaviour Assessment Questionnaire, *TRF* Teacher Report Form, *YSR* Youth Self Reports.

Statistics = *β* standardised parameter estimate, *OR* odds ratio, *R*^2^ percentage of variance explained, *r* weighted tetrachoric correlation. Effect sizes are not reported for studies that did not investigate both genetic and environmental transmission.

**Table 4:** Detailed characteristics of studies investigating offspring externalising behaviours (*N* = 36).

Offspring externalising behaviours								
Study	Design	Sample	Parental attribute (predictor)	Child attribute (outcome)	Control variables	Genetic overlap	Environmental transmission	G–E interplay
Bornovalova et al.^53^	Adoption	*SIBS* 402 adoptive, 204 biological families Age: 11–21 years	*Antisociality:* interview, SCI	*Maladaptive parenting:* self-report, PEQ *Marital discord:* self-report or marital status, MRS *Antisociality:* interview, SCI	Mother and father age, parental education, child ethnicity, child adoptive status, family-based clustering correction, child sex, age	Not studied	Adoptive maladaptive parenting and marital discord (but not antisociality) were associated with offspring disruptive behaviours	Parental antisociality & child disruptive behaviour disorders were associated in biological families, but not adoptive families. The authors interpret this as passive *rGE*, but it may be only indicative of genetic overlap
Kendler et al.^54^	Adoption	*Snr* 18,070 adoptees, and their biological (79,206) and adoptive (47,311) relatives Age: adoption until 20 years old	*Adoptive parental/sibling criminal behaviour risk:* composite score, criminal behaviour, alcohol use disorder (AUD), drug abuse, psychiatric illness, parental divorce *Biological parent/sibling criminal behaviour risk:* composite score, criminal behaviour, AUD, drug abuse, psychiatric illness, parental educational attainment (EA), maternal divorce, age at birth	*Criminal behaviour:* register-based, any conviction	Sex of the adoptee, birth year, age at first cohabitation with adoptive parents	The criminal behaviour of not-lived-with biological parent and siblings was associated with offspring criminal behaviour (OR = 1.5)	The criminal behaviour of adoptive family and siblings was associated with offspring criminal behaviour (OR range = 1.3–1.4)	No evidence of G × E interaction found
Lipscomb et al.^48^	Adoption	*EGDS* 233 families Age: 9 months to 6 years	*Adoptive parent over-reactive parenting:* self-report, PS *Birth parent self-regulation:* self-report, ATQ	*Externalising behaviour:* parent report, CBCL	Prenatal and obstetric complications, birth mother IQ, adoptive family SES, adoption openness, child age, sex, age of entry & time spent in early care	No, birth parent self-regulation did not predict offspring externalising behaviours	Yes, over-reactive adoptive parenting was associated with externalising behaviours (*β* = 0.14)	G×E: low birth parent self-regulation & exposure to early care-centre × over-reactive parenting: more externalising problems
Kendler et al.^55^	Multiple parenting relationships design	*Snr* 2,111,074 intact, 155,121 not-lived-with father, 10,194 not-lived-with mother, 107,163 stepfather, 17,637 stepmother, 10,038 adoptive families Age: 15+	*Criminal behaviour:* Swedish Crime register	*Criminal behaviour:* Swedish Crime register	Criminal behaviour status of all other relevant biological and step-parents	Yes, criminal behaviour of not-lived-with biological parents was correlated with offspring criminal behaviour (HR = 1.56)	Yes, criminal behaviour of adoptive or step-parent was correlated with offspring criminal behaviour (HR = 1.28)	
Kendler et al.^8^	Triparental family design	*Snr* 41,360 triparental families (mother, not-lived-with biological father, stepfather) Age: 15+	*Criminal behaviour:* Swedish Crime register	*Criminal behaviour:* Swedish Crime register		Yes, criminal behaviour of not-lived-with biological parents was correlated with offspring criminal behaviour (HR = 1.46)	Yes, criminal behaviour of adoptive or step-parent was correlated with offspring criminal behaviour (HR = 1.30)	
Hyde et al.^52^	Adoption	*EGDS* 561 families Age: 18–27 months	*Adoptive mother positive reinforcement:* observation *Birth mother antisocial behaviour:* self-report, DIS	*Externalising behaviours:* maternal report, CBCL *Callous - unemotional behaviours* *Oppositional behaviours* *Attention-deficit behaviours*	Child sex, openness/contact in the adoption, perinatal risk index	Birth mother antisocial behaviour predicted offspring callous–unemotional behaviours (*β* = 0.16), but not oppositional or attention-deficit behaviours	Adoptive mother positive reinforcement was protective against callous–unemotional (*β* = −0.19) and oppositional (*β* = −0.15), but not attention-deficit behaviours	G × E: high birth mother antisociality × low adoptive mother positive reinforcement: callous–unemotional behaviours
Stover et al.^49^	Adoption	*EGDS* 361 families Age: 9 months to 6 years	*Marital hostility:* self & spouse-report, BARS *Hostile parenting:* self-report, IFIRS *Birth mother antisociality*: self-report, composite score, delinquency (EYQ), substance use (CIDI), antisocial behaviour (CDIS)	*Aggression:* parent report, CBCL	Adoption openness	No, birth mother antisociality was not associated with offspring aggression	Adoptive parent hostile parenting and marital hostility were associated with offspring aggression (*β* range = −0.5 to 0.09)	
Reuben et al.^50^	Adoption	*EGDS* 361 families Age: 26 months to 7 years	*Warm parenting:* self-report, IFIRS *Over-reactive parenting:* self-report, PS *Birth mother externalising problems:* self-report, composite score, delinquency (ESBQ), novelty seeking (TCI) and drug dependence	*Externalising behaviour:* teacher-report, TRF *Effortful control:* shape Stroop task and gift delay task, the composite score	Prenatal risk and obstetric complications, adoption openness, birth mother externalising problems, child sex	No, birth mother externalising problems did not predict offspring externalising behaviour or effortful control	Adoptive maternal warm parenting (but not paternal, or over-reactive parenting) was associated with offspring externalising behaviours (*β* = −0.18), and this association was moderated by offspring effortful control	
Marceau et al.^51^	Adoption	*EGDS* 561 families Age: 4.5–8 years	*Adoptive parent warmth and hostility:* self-report, IWHS *Birth mother substance use during pregnancy:* study design cannot distinguish G and E effects *Birth mother internalising & externalising problems:* composite score, number of symptoms, diagnoses, age of onset, first degree relatives with psychopathology	*Conduct problems:* maternal report, Preschool Age Psychiatric Assessment	Adoption openness, child sex and earlier externalising problems	Birth mother externalising and internalising problems were associated with fewer conduct problems in boys (*β* range = −0.09 to −0.15) but not girls	Adoptive parent warmth and hostility were not associated with offspring conduct problems after controlling for earlier externalising problems	G × E: birth mother externalising problems × adoptive parent warmth and hostility (boys only)
Marceau et al.^41^	Adoption	*EGDS* 361 families Age: 9 months to 6 years	*Over-reactive parenting:* self-report, PS *Birth mother risk:* self-report, composite score, substance use, depression (BDI) and anxiety (BAI)	*Externalising behaviours:* parent report, CBCL	Adoption openness	No, birth mother risk did not predict offspring externalising behaviours (effect size not clear)	Yes, maternal (but not paternal) over-reactive parenting predicted offspring internalising behaviours (effect size not clear)	
McAdams et al.^19^	Adoption, children-of-twins	*Adoption: EGDS* *361 families* Age: 4.5–7 years *CoT: TOSS* 287 MZ and 489 DZ twin families Age: 11–22 years	*Adoptive & birth parent depression:* self-report, BDI *Depressive symptoms (CoT sample):* self-report, CES-D	*Externalising problems (adoption sample):* parent report, CBCL *Externalising problems (CoT sample):* mother, father and self-report, CBCL	Adoption sample: Obstetric complications, adoption openness CoT sample: twin sex, age	Adoption sample: Birth mother depressive symptoms predicted externalising problems at age 4.5 and 7 (*β* range = 0.13–0.16), but not age 6 CoT sample: No shared genetic effects between parental depression and offspring externalising problems	Adoption sample: No, adoptive parent depression did not predict subsequent offspring externalising problems CoT sample: Yes, after accounting for genetic relatedness, parental depression was associated with offspring externalising problems (effect size not clear)	Evocative *rGE*: birth mother depression predicted child externalising problems, which predicted adoptive parent depression
Roos et al.^57^	Adoption	*EGDS* 293 families Age: 6–7 years	*Adoptive & birth mother internalising symptoms:* self-report, composite score, BAI and BDI *Adoptive mother uninvolved parenting:* self-report, APQ *Adoptive & birth mother processing speed*: Stroop colour-word naming task	*Externalising-only problems:* parent report, CBCL *Co-occurring internalising and externalising problems:* parent report, CBCL	Child sex, child age, adoption openness, obstetric complications	Birth mother internalising symptoms and processing speed did not predict externalising-only symptoms, but maternal processing speed was associated with co-occurring symptoms (OR = 1.88)	Adoptive parent internalising symptoms, uninvolved parenting, and processing speed did not predict externalising-only problems, but uninvolved parenting was associated with co-occurring symptoms (OR = 7.91)	G × E: adoptive mother high internalising symptoms x inherited risk of slow processing speed: co-occurring symptoms
Grabow et al.^20^	Adoption	*EGDS, EPoCH* 541 adoptive mother–child pairs, 126 biological mother-biological child pairs Age: 7 years	*Maternal trauma frequency:* repeated self-report, mean score, NLES *Adoptive & birth mother depressive symptoms:* self-report, BDI	*Externalising behaviours:* parent report, CBCL, age 7	EPoCH: timing of maternal trauma, SES, child sex EGDS: Perinatal risk, adoption openness, SES, child sex	Yes, birth mother depression predicted adopted-away offspring externalising behaviours (*β* = 0.22)	Adopted mother depression predicted offspring externalising behaviours (*β* = 0.40), and mediated the relationship between maternal trauma and offspring externalising behaviours	
Gjerde et al.^24^	Sibling comparison	*MoBa* 11,599 families with 17,830 full siblings Age: 6 months to 5 years	*Maternal depression:* self-report, SCL	*Externalising problems:* maternal report, CBCL	Maternal parity, maternal EA, child age, child sex	Not studied	Children exposed to concurrent maternal depression had more externalising symptoms than their unexposed siblings, but perinatal maternal symptoms had no effect	
Hannigan et al.^21^	Multiple children-of-twins and siblings	*MoBa* 22,195 mothers and 25,299 children Age: 18–60 months	*Maternal depressive symptoms:* self-report, SCL	*Externalising problems:* maternal report, CBCL	Prenatal analyses: adjusted for concurrent depression	Yes, shared genetic effects between maternal depression and offspring externalising problems explained 37% of the variance (*R*^2^) in offspring externalising problems	No, after accounting for genetic relatedness, maternal depression was not associated with offspring externalising problems	
Gjerde et al.^22^	Multiple children-of-twins and siblings	*MoBa* 22,316 mothers and 35,589 offspring Age: 1.5 to 5 years	*Concurrent maternal depression symptoms:* self-report, SCL	*Behavioural problems:* maternal report, CBCL	Child sex, maternal age	Yes, there were shared genetic effects between maternal depression and offspring behavioural problems (*R*^2^ = 14.2–29.3%)	Yes, after accounting for genetic relatedness, maternal depression was associated with offspring behavioural problems (*R*^2^ = 0.4–1.3%)	
Hails et al.^25^	Adoption	EGDS 561 families Age: 9 months to 6 years	*Adoptive parent depression:* self-report, BDI-II *Birth mother internalising symptoms:* self-report, CIDI	*Externalising symptoms:* parent and teacher report, CBCL and TRF	Adoption openness, prenatal risk and obstetric complications, infant negative emotionality	Yes, the birth mother’s internalising symptoms predicted parent (but not teacher) rated offspring externalising symptoms (*β* = 0.11)	Adoptive maternal (but not paternal) depression predicted offspring externalising symptoms (*β* = 0.11)	
Eilertsen et al.^56^	Children-of-twins and siblings	*MoBa* 17,070 extended-family units Age: 5 years	*Parental prenatal depression symptoms:* self-reported at pregnancy week 30 for mothers, week 17 for fathers, Symptom Checklist	*ADHD symptoms:* maternal report, CPRS		Yes, there were shared genetic effects between parental depression and offspring ADHD symptoms (*β* = 0.42)	After accounting for genetic relatedness, maternal (but not paternal) prenatal depression was associated with offspring ADHD symptoms (*β* = 0.07)	
Gjerde et al.^31^	Sibling comparison	*MoBa* 17,724 offspring and 11,553 mothers Age: 1.5–5 years	*Maternal anxiety symptoms:* self-report, SCL	*Externalising problems:* maternal report, CBCL	Child age, sex, maternal depressive symptoms, parity and education	Not studied	No difference in externalising problems between exposed children and their unexposed siblings	
Samek et al.^64^	Adoption	*SIBS* 525 adopted and 323 biological offspring Age: 16.5 years and older	*Parent–child relationship quality:* offspring report, PEQ *Alcohol and tobacco use:* mother & father report, composite score, SAM and CSUA	*Externalising behaviours:* latent factor based on antisocial behaviour (self-report, SCI), risky sexual behaviour (self-report, LEI) & alcohol and tobacco use (self-report, SAM)	Child age, sex, ethnicity, SES	Not studied	Adoptive parent relationship quality with child (but not alcohol and tobacco use) was associated with offspring externalising behaviours	The study states that it provides evidence against passive *rGE*, but in fact the adoption-at-birth design excludes passive rGE
Elam et al.^61^	Adoption	*EGDS* 316 families Age: 27 months to 4.5 years	*Adoptive parent hostility:* self-report, IFIRS	*Disruptive peer behaviour:* parent report, PIPPS	Prenatal risk and obstetric complications, adoption openness	Not studied	Adoptive mother–child and father–child hostility predicted offspring disruptive peer behaviours	Evocative *rGE*: birth mother low behavioural motivation predicted toddler low social motivation, which predicted adoptive parent–child hostility
Marceau et al.^59^	Extended children-of-twins	*NEAD, TOSS* 408 twin/sibling pairs, 854 twin families Age: 11–22 years	*Parental knowledge:* mother, father and self-report, composite score, CMS	*Externalising problems:* mother, father, and self-report, composite score, ZBPI (NEAD sample), CBCL (TOSS sample)	Age, sex, age difference between non-twin siblings and cousins	No, there were no shared genetic effects between parental knowledge and offspring externalising problems	Yes, after accounting for genetic relatedness, parental knowledge was associated with offspring externalising problems (effect size not clear)	No passive or evocative *rGE* found
Guimond et al.^65^	Sibling comparison	*QNTS* 164 twin pairs Age: 13–14 years	*Perceived maternal support and negativity:* child report, NRI	*Delinquent behaviours:* self-report, S-RDQ	Genetically controlled analyses using MZ twin-difference score	Not studied	No, perceived maternal support and negativity were not associated with offspring delinquent behaviours	No evocative *rGE*, but child-to-parent effects found
Plamondon et al.^62^	Sibling comparison	*KFP* 397 families, 920 children Age: 1.5–4 years	*Maternal negativity:* self-report, NLSCY	*Child disruptive behaviour:* mother and father report, mean score, OCHS	Maternal EA, child sex and child age	Not studied	Exposed children showed more disruptive behaviours than their unexposed sibling	
Trentacosta et al.^60^	Adoption	*EGDS* 561 families Age: 18 months to 4.5 years	*Adoptive parent harsh parenting:* self-report, PS *Inherited risk:* self-report, birth mother fearlessness (BISS) and interpersonal affiliation (HAS-PP)	*Callous– unemotional behaviours:* parent report, CBCL	Pregnancy and obstetric complications, adoption openness, child gender, oppositional behaviour	No difference in callous–unemotional behaviours in children with high or low inherited risk	Adoptive parent harsh parenting was associated with callous–unemotional behaviours at 54, but not at 27 months (*β* range = 0.12–0.15)	G × E: high inherited risk (high birth mother fearlessness and low affiliation) × adoptive father harsh parenting: callous–unemotional behaviours
Ellingson et al.^71^	Sibling comparison	*CNLSY* 10,251 children of 4,827 mothers Age: 4–14 years	*Smoking during pregnancy:* self-report, mean number of packs smoked per day	*Disruptive behaviour:* maternal report, BPI	Maternal age at birth, EA, intelligence, delinquency, offspring sex, birth order, ethnicity, household income, geographic location	Not studied	No difference in disruptive behaviours between exposed children and their unexposed siblings	
Kuja-Halkola et al.^67^	Sibling comparison, children-of-twins	*Snr* 2,754,626 children Age: up to 20 years	*Maternal smoking during pregnancy:* self-report	*Criminality:* national crime register, any conviction	Maternal age at childbirth, child sex, birth year	Yes, there were shared genetic effects between maternal smoking during pregnancy and offspring criminality (effect size not clear)	No, exposed children did not differ from their unexposed siblings, and after accounting for genetic relatedness, maternal smoking was not associated with offspring criminality	
Kendler et al.^66^	Adoption	*Snr* 1010 intact, 9944 triparental, 56,906 not-lived-with father, 6141 not-lived-with mother, 25,027 stepfather, 5049 stepmother, 837 adoptive families Age: not reported	*Drug abuse:* Swedish medical registers, the Suspicion Register, the Crime Register, drug-related driving offenses, and the Prescribed Drug Register	*ADHD:* Hospital Discharge Register, the Outpatient Care Register, and the Prescribed Drug Register		Yes, birth parent drug abuse was associated with offspring ADHD (HR range = 2.06–2.48)	No, adoptive or step-parent drug abuse was not associated with offspring ADHD	
Obel et al.^73^	Sibling comparison	*DNR* Families of 17,381 children with ADHD Age: 3 years to diagnosis	*Maternal smoking during pregnancy:* self-report	*ADHD:* diagnosis of hyperkinetic disorder, or prescription of ADHD medication for at least 6 months	Maternal age, parity, child sex, year of birth	Not studied	No difference in ADHD diagnosis between exposed or unexposed siblings	
Knopik et al.^72^	Sibling comparison	*MO-MATCH study* 173 mothers and their offspring Age: 10–12 years	*Smoking during pregnancy:* maternal report, MAGIC-PC	*ADHD symptoms:* parent and teacher-report, CRS	Maternal marital status at birth, food stamp usage at delivery, exposure to paternal smoking during pregnancy, childbirth order, sex	Not studied	Exposed children had more parent-reported (but not teacher-reported) ADHD symptoms than their unexposed siblings	
Estabrook et al.^70^	Sibling comparison	*MIDS* 299 families Age: 3–18 years	*Maternal smoking during pregnancy:* self-report	*ADHD:* SBSC *Oppositional Defiant Disorder (ODD):* SBSC *Conduct Disorder (CD):* SBSC	Offspring age, sex, parental history of antisocial behaviour (Antisocial Behaviour Questionnaire)	Not studied	Exposed children were more likely to show oppositional defiant disorder and conduct disorder (but not ADHD) than their unexposed siblings	
Eilertsen et al.^74^	Sibling comparison	*MoBa* 16,407 mothers and 34,283 children Age: 5 years	*Maternal alcohol use during pregnancy:* AUDIT-C	*ADHD symptoms:* maternal report, revised CRS and CBCL *ADHD diagnosis:* diagnosis	Parental EA, parental income, maternal smoking during pregnancy, children’s birth order, gender	Not studied	Exposed children had more ADHD symptoms (according to CPGS-R, but not CBCL) than their unexposed siblings, but did not differ in ADHD diagnosis	
Lund et al.^47^	Sibling comparison	*MoBa* 14,639 mothers, 25,744 children Age: 1.5–5 years	*Maternal alcohol consumption during pregnancy:* self-report, AUDIT-C	*Behavioural problems:* maternal report, CBCL *Attention problems* *Aggressive behaviours*	Parity, unplanned pregnancy, daily smoking, pre-pregnancy abstinence from alcohol	Not studied	Exposed children were more aggressive, but did not have more attentional problems, than their unexposed sibling	
Pingault et al.^13^	Within-family PGS: genetic sensitivity analysis	*TEDS* 3663 to 4693 individuals Age: 8–16 years	*Maternal EA:* self-report, eight levels	*ADHD:* maternal, report, mean score, CRS-Revised	Sex, age and ten principal components of ancestry, PGS for EA and ADHD	Yes, the association between maternal EA and offspring ADHD decreased after adjusting for EA and ADHD PGS (from *β* = −0.13 to *β* = −0.11)	Under a twin-heritability scenario, the association between maternal EA and offspring ADHD was expected to be null if EA and ADHD PGS captured all heritability	
Torvik et al.^45^	Children-of-twins and siblings	*MoBa* 34,958 children Age: 8 years	*Educational attainment:* self-report, highest level completed	*ADHD symptoms:* maternal report, RSDBDs		Yes, there were shared genetic effects between parental EA and offspring ADHD symptoms (effect size not clear)	Yes, after accounting for genetic relatedness, parental EA was associated with offspring ADHD (effect size not clear)	
de Zeeuw et al.^75^	Within-family PGS: genetic nurture (transmitted/non-transmitted method)	*NTR* 5900 offspring, 2649 families Age: 10–12, 25–64 years	*Genetic transmission:* effect of transmitted alleles PGS for EA and ADHD *Genetic nurture:* effect of non-transmitted alleles PGS for EA and ADHD	*ADHD symptoms:* parent and teacher report, at-home and at-school symptoms, CBCL and TRF	Sex, year of birth (for EA), the interaction between sex and year of birth (for EA), ten principal components, genotyping platform	EA and ADHD PGS based on transmitted parental alleles were associated with offspring ADHD symptoms at home and at school (*R*^2^ = 0.8–2%)	EA and ADHD PGS based on non-transmitted parental alleles were not associated with offspring ADHD symptoms at home and at school	

*G–E* gene–environment, *G×E* gene–environment interaction, *rGE* gene–environment correlation.

Design = *CoT* children-of-twins, *PGS* polygenic scores.

Samples = *CNLSY* Children of the National Longitudinal Survey of Youth, *EGDS* Early Growth and Development Study, *Dnr* Danish national registers, *EPoCH* Early Parenting of Children study, *MIDS* Midwest Infant Development Study, *KFP* Kids, Families, and Places Study, *MoBa* Norwegian Mother Father and Child Study, *MO-*MATCH Missouri Mothers and Their Children Study, *NEAD* Nonshared Environment in Adolescent Development Study, *NTR* Netherlands Twin Register, *QNTC* Quebec Newborn Twin Study, *SIBS* Sibling Interaction and Behaviour Study, *Snr* Swedish national registers*, TEDS* Twins Early Development Study, *TOSS* Twin Offspring Study of Sweden.

Measures = *APQ* Alabama Parenting Questionnaire, *ATQ* Adult Temperament Questionnaire, *AUDIT-C* Alcohol Use Disorder Identification Test-Consumption, *BAI* Beck Anxiety Inventory, *BARS* Behaviour Rating Scale, *BDI* Beck Depression Inventory, *BISS* Behavioural Inhibition System scale, *BPI* Behaviour Problem Index, *CBCL* Child Behaviour Checklist, *CDIS* Computerised Diagnostic Interview Schedule, *CES-D* Centre for Epidemiological Studies Depression Scale, *CIDI* Composite International Diagnostic Instrument, *CMS* Child Monitoring Scale, *CRS* Conner’s Rating Scale, *CSUA* Computerised Substance Use Assessment, *DIS* Diagnostic Interview Schedule*, ESBQ* Elliott Social Behaviour Questionnaire, *EYQ* Elliott Youth Questionnaire, *HAS-PP* Harter Adult Self-Perception Profile scale, *IFIRS* Iowa Family Interaction Rating Scales, *IWHS* Iowa Warmth and Hostility Scales, *LEI* Life Events Interview, *MAGIC-PC* Missouri Assessment of Genetics Interview for Children–Parent on Child, *MRS* Marital Relationship Questionnaire, *NLES* Negative Life Events Scale, *NRI* Network of Relationships Inventory, *NLSCY* negativity scale from the National Longitudinal Survey of Children and Youth, *OCHS* conduct disorder-aggression scale from the Ontario Child Health Study, *PEQ* Parental Environment Questionnaire, *PIPPS* Penn Interactive Peer Play Scale, *PS* the Parenting Scale*, RSDBD* Rating Scale for Disruptive Behaviour Disorders, *SAM* Substance Abuse Module, *SBSC* Stony Brook Symptom Checklist, *SCI* Structured Clinical Interview for DSM-III-R, *SCL* Symptoms Checklist, *S-RDQ* Self-Report Delinquency Questionnaire, *TCI* Temperament Characteristic Inventory, *TRF* Teacher Report Form, *ZBPI* Zill Behaviour Problems Inventory.

Statistics = *β* standardised parameter estimate, *OR* odds ratio, *HR* hazard ratio, *R*^2^ percentage of variance explained. Effect sizes are not reported for studies that did not investigate both genetic and environmental transmission.

**Table 5:** Detailed characteristics of studies investigating offspring educational attainment and cognition (*N* = 21).

Offspring educational attainment and cognition								
Study	Design	Sample	Parental attribute (predictor)	Child attribute (outcome)	Control variables	Genetic overlap	Environmental transmission	G–E interplay
Kendler et al. ^124^	Adoption (siblings-reared-apart)	*Snr* 436 sibships, one member reared by biological, other by adoptive parents Age: 18–20 years	*EA:* highest education achieved by both parents, five-point-scale	*IQ:* Military Conscription Register, standardised test	Clustering of siblings within biological families	Not studied	Yes, adoptive parent EA predicted offspring IQ	
Conley et al.^82^	Within-family PGS: genetic sensitivity analysis, and genetic nurture (statistical control method)	*FHS, HRS* 6186 individuals from 4867 households Mean age: 39.49 years (FHS), 68.17 years (HRS)	*Parental education* *Genetic transmission:* effect of parental EA PGS *Genetic nurture:* effect of parental EA PGS, after adjusting for child EA PGS	*EA:* self-report, highest grade completed	Child sex, age	Yes, parental EA PGS predicted offspring EA (effect size not clear)	Genetic sensitivity analysis: After controlling for offspring EA PGS, parental EA was still associated with offspring EA. Genetic nurture: no evidence of genetic nurture as parental EA PGS was not associated with offspring EA after controlling for offspring EA PGS (effect size not clear)	No G × E interaction found between maternal EA and offspring PGS
Ayorech et al.^79^	Extended twin, within-family PGS	*TEDS* Twin analyses: 6105 twin pairs PGS analyses: 5825 individuals Age: 18 years	*EA (extended twin):* self-reported highest qualification *Genetic transmission (within-family PGS):* effect of parental EA PGS	*EA:* self or parent report, A levels qualification *Intergenerational EA (extended twin):* similarity between parental and offspring EA, two levels *Intergenerational EA (within-family PGS):* similarity between parental and offspring EA, four levels	PGS analyses: previous school performance (GCSE grades)	Twin analyses: yes, additive genetic effects underlying intergenerational EA were found (*R*^2^ = ~50%) PGS analyses: yes, parental EA PGS was associated with intergenerational EA	Twin analyses: yes, shared environmental effects underlying intergenerational EA were found (*R*^2^ = ~40%) PGS analyses: Not studied	
Scheeren et al.^90^	Adoption	*NLnr* 1792 adopted children, 424,928 biological children Age: 15 years	*EA:* register-based, highest education level *Parental income:* yearly household income	*EA:* level of enrolment in secondary school, four levels	Father and mother year of birth, family structure, number of children in the household, observation year, adoption age, country of adoption, gender	Not studied	Adoptive parents’ income (but not EA) predicted offspring EA	Passive *rGE*: family income was more strongly associated with offspring EA in biological families than adoptive families
Bates et al.^14^	Within-family PGS: genetic nurture (transmitted/non-transmitted method)	*BATS* 2,335 children and their genotyped parents Age: 17 years	*Genetic nurture:* effect of EA PGS based on non-transmitted alleles *SES:* ASI-2006	*EA:* Queensland Core Skills Test	Sex, age at test, offspring EA PGS	Not studied	PGS for EA based on non-transmitted alleles were associated with offspring EA, but this relationship disappeared after adjusting for parental SES	No G × E interaction found between PGS and SES
Belsky et al.^86^	Within-family PGS: genetic nurture (statistical control method)	*E-RISK, NLAAH* 1574 & 5526 individuals Age: 18 years, late 20 s to early 30 s	*Genetic nurture:* effect of parental EA PGS, after adjusting for child EA PGS	*EA:* GCSE attainment; four levels	Genetic principal components	Not studied	Yes, parental EA PGS was associated with offspring EA after adjusting for offspring EA PGS	Passive *rGE*: individuals with higher PGS grew up in better-educated households
Kong et al.^83^	Within-family PGS: genetic nurture (transmitted/non-transmitted method)	*deCODE* 21,637 probands with at least one genotyped parent Age: not reported	*Genetic transmission:* effect of EA alleles PGS based on transmitted alleles *Genetic nurture:* effect of EA PGS based on non-transmitted alleles	*EA*	Sex, year of birth, the interaction between sex and year of birth, 100 principal components	Yes, EA PGS based on transmitted parental alleles was associated with offspring EA (direct effect explained 70% of the overall observed effect of EA PGS)	Yes, EA PGS based on non-transmitted parental alleles was associated with offspring PGS (genetic nurture explained explaining 22.4% of the overall effect of EA PGS)	
Liu et al.^84^	Within-family PGS: genetic nurture (statistical control method)	*FHS, HRS* 8639 individuals from three generations and 9342 individuals over age 50 Age: not reported	*Genetic transmission (FHS sample):* effect of parental EA PGS *Genetic nurture (FHS sample):* effect of parental EA PGS, after adjusting for child PGS *EA (HRS sample):* self-report, years of education	*EA* *FHS:* self-report, years of education completed *HRS:* parent report	7 principal components HRS sample: child’s EA PGS	Yes, parental EA PGS was associated with offspring EA (FHS sample; *β* = 0.345), and offspring EA PGS attenuated the association between parental and offspring EA (HRS sample; from *β* = 0.314 to *β* = 0.292)	Yes, parental EA PGS was associated with offspring EA, after adjusting for offspring EA PGS (*β* = 0.076)	
Young et al.^17^	Relatedness disequilibrium regression	*deCODE* 12,035 individuals who had parents and grandparents genotyped Age: not reported	*Genetic nurture:* estimated variance in offspring trait explained by parental genes acting indirectly via the environment	*Educational attainment:* self-report, number of years of schooling	Sex, year of birth	Not studied	Yes, after accounting for shared genetic effects, parental genes explained variance in offspring EA	
Pingault et al.^13^	Within-family PGS: genetic sensitivity analysis	*TEDS* 3663–4693 individuals Age: 8–16 years	*Maternal EA:* self-report, eight levels	*EA:* mean of three standardised tests	Sex, age and ten principal components of ancestry, PGS for EA	Yes, association between maternal EA and offspring EA decreased after adjusting for EA PGS (from *β* = 0.40 to 0.33)	Under a twin-heritability scenario, the association between maternal and offspring EA was expected to be null if EA PGS captured all heritability	
Bates et al.^15^	Within-family PGS: genetic nurture (transmitted/non-transmitted method)	*BATS* 2335 children and their genotyped parents Age: 17 years	*Genetic nurture:* effect of parental EA PGS based on non-transmitted alleles *SES:* ASI-2006	*EA:* Queensland Core Skills Test	Sex, age at test, offspring EA PGS	Not studied	PGS for EA based on non-transmitted alleles were associated with offspring EA, but this relationship disappeared after adjusting for parental SES	No G × E interaction found between PGS and SES
Willoughby et al.^88^	Within-family PGS: genetic nurture (statistical control method)	*MCTFR* 1223 families, 2446 offspring Age: varied	*Genetic nurture:* effect of parental EA PGS, on top of child EA PGS *SES:* composite score, family income, parent education level, parent occupation level *Parental IQ:* WIS	*Years of education:* self-report, mean age 29 *High-school grade-point-average:* self-report, age 17 *IQ:* WIS, mean age 14.4	Height and BMI used as negative controls	Not studied	Yes, parental EA PGS was associated with offspring EA traits after adjusting for offspring EA PGS, and this association was mediated by parental SES and IQ	
Armstrong-Carter et al.^89^	Within-family PGS: genetic nurture (statistical control method)	*BiBs* 2077 mother–child dyads Age: 7 years	*Genetic nurture*: effect of maternal EA PGS, after adjusting for child EA PGS *Maternal health:* composite score, self-reported mental health, smoking, indirect smoke exposure, alcohol and drug use, vitamin use, sleep problems, and BMI *SES*: composite score, self-reported education, cohabitation status, employment, maternity leave, governmental benefits, perceived financial difficulty, and governmental index of neighbourhood-level deprivation	*Academic performance:* standardised national exam	Child EA PGS, maternal age, first ten principal components	Not studied	Yes, maternal EA PGS was associated with offspring academic performance, after adjusting for offspring EA PGS, and this association was mediated by maternal health and SES during pregnancy	
Borriello et al.^80^	Adoption	*EGDS* 195 families Age: 7 years	*Mathematical achievement:* standardised scores on the mathematics fluency subtest of WJ-III	*Mathematical achievement:* standardised scores on the mathematics fluency subtest of the WJ-III	Obstetric complications, adoption opennness, parent education level, non-mathematical cognitive skills	Yes, birth parent and offspring mathematic achievement were correlated (*β* = 0.17)	Yes, paternal (but not maternal) mathematic achievement was correlated with adopted-offspring mathematical achievement (*β* = 0.15)	No G × E interaction found
Domingue et al.^85^	Adoption (PGS study)	*WLS* 855 adopted and 20,939 biological offspring Age: not reported	*Genetic transmission:* association between parental EA PGS and EA of biological offspring *Genetic nurture:* association between parental EA PGS and EA of adoptive offspring	*Educational attainment:* parent-reported, highest grade of school attended	Child sex, age, ten principal components	Yes, parental EA PGS was associated with EA of biological offspring (effect size not clear)	Yes, parental EA PGS was associated with EA of adoptive offspring (effect size not clear)	Passive *rGE* implied: higher association in biological families than adoptive families
de Zeeuw et al.^75^	Within-family PGS: genetic nurture (transmitted/non-transmitted method)	*NTR* 5900 offspring from 2649 families Age: 10–12, 25–64 years	*Genetic transmission:* effect of EA and ADHD ADHD PGS based on transmitted alleles *Genetic nurture:* effect of EA and ADHD PGS based on non-transmitted alleles	*Childhood academic achievement:* nationwide standardised test at age 12 *Adult EA:* self-report, highest degree; four levels	Sex, birth year (EA), interaction between sex and birth year (EA), ten principal components, genotyping platform	EA PGS based on transmitted parental alleles were associated with offspring academic achievement in childhood and EA in adulthood (*R*^2^ = 5.7–7.6%) but there was no association with ADHD PGS	EA PGS based on non-transmitted parental alleles were associated with offspring EA in adulthood (*R*^2^ = 1.7%), but not academic achievement in childhood (which was also not associated with non-transmitted PGS for ADHD)	
Halpern-Manners et al.^81^	Adoption	*EGDS* 340 families Age: first-graders (6–7 years)	*Adoptive and birth parent education attainment*: self-report, highest level of education completed by adoptive or birth parents	*Early educational achievement*: WJ-III	Obstetric complications, adoption opennness, child sex, child and adoptive parents’ ethnicity, adoptive parents’ age, type of adoption agency	Yes, birth parent EA was associated with offspring EA (effect size not clear)	Yes, adoptive parent EA was associated with offspring EA (effect size not clear)	No G × E interaction
Torvik et al.^45^	Children-of-twins and siblings	*MoBa* 34,958 children Age: 8 years	*Educational attainment:* self-report, highest level completed	*Academic problems:* maternal report, three-point scale		Yes, there were shared genetic effects between parental EA and offspring academic problems (effect size not clear)	Yes, after accounting for genetic relatedness, parental EA was associated with offspring academic problems (effect size not clear)	
Ellingson et al.^71^	Sibling comparison	*CNLSY* 10,251 children of 4827 mothers Age: 4–14 years	*Smoking during pregnancy:* self-report, mean number of packs smoked per day	*Cognitive functioning:* PPVT-R (math, reading and reading Recognition subtests) and digit span test	Maternal age at birth, EA, intelligence, delinquency, offspring sex, birth order, ethnicity, household income, geographic location	Not studied	Exposed children had poorer reading recognition than their unexposed siblings, but there were no other group differences	
Kuja-Halkola et al.^67^	Sibling comparison, children-of-twins	*Snr* 2,754,626 children Age: up to 20 years	*Maternal smoking during pregnancy:* self-report	*Academic achievement*: class 9 records *General cognitive ability*: Military Conscription Register, nine levels	Maternal age at childbirth, child sex, birth year	Yes, there were shared genetic effects between maternal smoking during pregnancy and offspring EA traits (effect size not clear)	No, exposed children did not differ from their unexposed siblings, and after accounting for genetic relatedness, maternal smoking was not associated with offspring EA traits	
Wertz et al.^78^	Within-family PGS: genetic nurture (statistical control method)	*E-RISK* 860 mothers and their children Age: 18 years	*Genetic nurture:* effect of maternal EA PGS, after adjusting for child PGS *Parenting behaviour:* mother, child and interviewer report, *cognitive stimulation, warmth and sensitivity, household chaos, and safety and tidiness of the family home*	*EA:* self-report, highest educational attainment, 18 years	Sex, first ten principal components, offspring EA PGS	Yes, controlling for offspring EA PGS attenuated the association between parenting behaviours and offspring EA (from *β* range = 0.33–0.52 to β range = 0.30–0.48)	Genetic nurture: yes, maternal EA PGS was associated with offspring EA after adjusting for offspring EA PGS (*β* = 0.11), and this effect was mediated by parenting behaviours including cognitive stimulation, household chaos and a safe, tidy home (but not parental warmth)	Evocative *rGE*: mother and offspring PGS for EA predicted cognitive stimulation and warm, sensitive parenting

*G–E* gene–environment, *G* *×* *E* gene–environment interaction, *rGE* gene–environment correlation.

Design = *PGS* Polygenic scores.

Samples = *BATS* Brisbane Adolescent Twin Study, *BiBs* Born in Bradford study, *CNLSY* Children of the National Longitudinal Survey of Youth, *EGDS* Early Growth and Development Study, *deCODE* Icelandic Genealogy Database, *FHS* Framingham Heart Study, *HRS* Health Retirement Study, *MoBa* Norwegian Mother Father and Child Study, *MCTFR* Minnesota Centre for Twin and Family Research, *NLNR* Dutch national registers, *NLAAH* National Longitudinal study of Adolescent to Adult Health, *NTR* Netherlands Twin Register, *SNR* Swedish national registers*, TEDS* Twins Early Development Study, *WLS* Wisconsin Longitudinal Study.

Measures = *ASI* Australian Socioeconomic Index occupational status scale, *PPVT-R* Peabody Picture Vocabulary Test-Revised, *QCST* Queensland Core Skills Test, *WIS* Weschler Intelligence Scale, *WJ-III* Woodcock–Johnson Test of Achievement III.

Statistics = *β* standardised parameter estimate, *R*^2^ percentage of variance explained. Effect sizes are not reported for studies that did not investigate both genetic and environmental transmission.

**Table 6:** Detailed characteristics of studies investigating offspring substance use behaviours (*N* = 19).

Offspring substance use behaviours								
Study	Design	Sample	Parental attribute (predictor)	Child attribute (outcome)	Control variables	Genetic overlap	Environmental transmission	G–E interplay
McGue et al.^106^	Adoption	*SIBS* 409 adoption and 208 biological families Age: 10–28 years	*Drinking behaviour:* self-report, composite score, CSUA and SAM	*Drinking behaviour:* self-report, composite score, CSUA and SAM	Parent gender, and child gender	Not studied	Yes, adoptive parent drinking behaviour was associated with offspring drinking behaviour	Passive *rGE* implied: parent–offspring association was greater in biological pairs than adoptive pairs
Waldron et al.^94^	Children-of-twins	*MATCH, PACER* 1318 offspring of twin parents Age: 11–24 years	*Substance dependence:* self-report, SAGA *Parental separation:* study design cannot distinguish G and E effects	*Offspring substance involvement:* self-report, SAFA	Parent or offspring comorbid psychopathology, twin sex, twin age, twin EA, child sex, age	Substance dependence: yes, there were shared genetic effects between parental substance dependence and offspring substance involvement (effect size not clear)	Substance dependence: after accounting for genetic relatedness, parental substance dependence was not associated with offspring substance involvement with the exception of cannabis use which was associated with offspring smoking behaviour (effect size not clear)	
Kuja-Halkola et al.^67^	Sibling comparison, children-of-twins	*Snr* 2,754,626 children Age: up to 20 years	*Maternal smoking during pregnancy:* self-report	*Drug/alcohol misuse:* register-based, diagnosis, or drug-related conviction	Maternal age at childbirth, child sex, birth year	Yes, there were shared genetic effects between maternal smoking during pregnancy and offspring drug/alcohol misuse (effect size not clear)	No, exposed children did not differ from their unexposed siblings, and after accounting for genetic relatedness, maternal smoking was not associated with offspring drug/alcohol misuse	
Kendler et al.^95^	Adoption	*Snr* 18,115 adoptees, 171,989 not-lived-with parent, and 107,699 step-parent families Mean age: 33.9 years	*AUD:* Swedish Hospital Discharge Register, the Swedish Prescribed Drug Register, the Outpatient Care Register, the Primary Health Care Register, and the Swedish Crime and Suspicion Register	*AUD:* Swedish Hospital Discharge Register, the Swedish Prescribed Drug Register, the Outpatient Care Register, the Primary Health Care Register, and the Swedish Crime and Suspicion Register		Yes, birth parent AUD predicted offspring AUD (OR = 1.46)	Yes, adoptive parent AUD predicted offspring AUD (OR = 1.40)	No G × E interaction observed
Grant et al.^96^	Children-of-twins	*VET* 1828 offspring of male twin parents Age: not reported	*Parental alcohol or drug dependency:* diagnosis, DIS *Parental separation:* study design cannot distinguish G and E effects	*Alcohol involvement:* self-report, SAGA	Maternal alcohol dependency, heavy cannabis use, family income, child sex, age, history of psychiatric problems and traumatic life events, inattention, hyperactivity and oppositional defiant disorder	Substance dependency: yes, there were shared genetic effects between parental substance dependence and offspring alcohol involvement (effect size not clear)	Substance dependency: yes, after accounting for genetic relatedness, parental substance dependency was associated with offspring alcohol involvement (effect size not clear)	
Kendler et al.^8^	Triparental family design	*Snr* 41,360 triparental families (mother, not-lived-with biological father, and stepfather) Age: 15+	*Drug abuse:* medical registries, the Crime Register, the Suspicion Register, drug-related driving offences, and the Prescribed Drug Registe*r* *AUD:* medical and mortality registries, the Suspicion Register, the Crime Register, and the Prescribed Drug Register	*Drug abuse:* medical registries, the Crime Register, the Suspicion Register, drug-related driving offences, and the Prescribed Drug Registe*r* *AUD:* medical and mortality registries, the Suspicion Register, the Crime Register, and the Prescribed Drug Register		Yes, drug abuse and AUD registration of not-lived-with biological parents were correlated with offspring drug abuse and AUD (HR range = 1.84–2.45)	Yes, drug abuse or AUD registration of adoptive or step-parent correlated with offspring drug abuse or AUD (HR range = 1.27–1.99)	
Kendler et al.^98^	Triparental family design	*Snr* 2,111,074 offspring in intact families 155,121 not-lived-with father, 10,194 not-lived-with mother, 107,163 stepfather, 17,637 stepmother 10,038 adoptive families Age: 15+	*Drug abuse:* medical registers, the Crime Register, the Suspicion Register, and drug-related driving offences	*Drug abuse:* medical registers, the Crime Register, the Suspicion Register, and drug-related driving offences	Drug abuse status of all other relevant biological and step-parents	Yes, drug abuse behaviour of not-lived-with biological parents were correlated with offspring drug abuse (HR = 2.73)	Yes, drug abuse behaviour of adoptive or step-parent correlated with offspring drug abuse (HR = 1.79)	
Bidwell et al.^105^	Sibling comparison	*MO-MATCH* 173 mothers and their offspring Age: 7–15 years	*Smoking during pregnancy:* self-report, MAGIC-PC	*Substance use:* self-report, DUSI	Maternal age, marital status, EA, qualification for food stamps at the time of delivery, parental substance use outside of pregnancy, childbirth order, sex, exposure to paternal smoke during pregnancy	Not studied	No difference in substance use behaviours between exposed children and their unexposed siblings	
Kendler et al.^97^	Extended family design	*Snr* 38,373 offspring of not-lived-with fathers and 9711 offspring of step-fathers Age: 15+	*AUD:* medical registries, the Prescribed Drug Register, two or more convictions of drunk driving in the Crime register	*AUD:* medical registries, the Prescribed Drug Register, two or more convictions of drunk driving in the Crime register	AUD in the biological mother, and offspring sex	Yes, not-lived with father AUD (including age of registration, recurrence and number of AUD registrations) predicted offspring AUD (HR not reported)	Yes, stepfather AUD (including the number of registrations that occurred while co-offspring with offspring) predicted offspring AUD (HR = 1.03)	
Treur et al.^100^	Children-of-twins, within-family PGS: genetic sensitivity analysis	*NTR* CoT sample: 712 twins, 723 children PGS sample: 4072 individuals Age: not reported	*Smoking initiation (CoT sample):* self-report *Exposure to smoking (PGS sample):* offspring-reported exposure as a child (up to age 18)	*CoT sample smoking initiation:* self-report *PGS sample smoking behaviour:* self-report, smoking initiation and smoking heaviness	CoT: twin sex, twin age, child sex, age, family-based clustering correction PGS: sex, year of birth, ten principal components, family clustering correction	CoT sample: yes, there were shared genetic effects between parent and offspring smoking initiation (effect size not clear) PGS sample: not studied	CoT sample: yes, after accounting for genetic relatedness, parent smoking initiation was associated with offspring smoking initiation (effect size not clear) PGS sample: yes, after adjusting for smoking PGS, exposure to smoking during childhood was associated with smoking initiation (OR = 1.68)	G×E: high PGS for smoking initiation & heaviness × childhood exposure to smoking: smoking heaviness (no interaction for smoking initiation)
Maes et al.^103^	Extended twin	*V-30, A-25* 22,393 twins and their families Age: 18+	*Smoking initiation:* self-report	*Smoking initiation:* self-report	Age	Not studied	There were shared environmental effects underlying parent–offspring similarity in smoking initiation (negative cultural transmission)	Passive *rGE*: negative covariance between additive genetic effects and parental smoking
Kendler et al.^99^	Multiple parenting relationships design	*Snr* 2,111,074 intact, 41,360 triparental, 113,762 not-lived-with father, 10,194 not-lived-with mother, 65,803 stepfather, 17,637 stepmother, 10,038 adoptive families Age: not reported	*Drug abuse:* medical and mortality registries, the Suspicion and Crime registers, drug-related driving offences, and the Prescribed Drug Register	*Drug abuse:* medical and mortality registries, the Suspicion and Crime registers, drug-related driving offences, and the Prescribed Drug Register		Yes, drug abuse behaviour of not-lived-with biological parents were correlated with offspring drug abuse (*r* range = 0.13–0.19)	Yes, drug abuse behaviour of adoptive or step-parent correlated with offspring drug abuse (*r* range = 0.06–0.09)	
Kendler et al.^11^	Matched-pairs case–control	*Snr* 65,006 parent–offspring, sibling, and cousin pairs Age: 19–23 years	*Drug abuse:* medical registers, the Crime Register, the Suspicion Register, and drug-related driving offences	*Drug abuse:* medical registers, the Crime Register, the Suspicion Register, and drug-related driving offences in offspring whose parents had a drug abuse incident 1–3 years ago	Control parent–child pairs matched on sex, parent and child year of birth, country of birth, SES, number of lifetime drug abuse registrations, medical or criminal registration, parental EA	Not studied	Yes, exposed offspring were at increased risk of drug abuse than matched control offspring who were unexposed to parental drug registration	
Kendler et al.^9^	Multiple parenting relationships design	*Snr* 475,000 parent–offspring pairs Age: 15 and over	*Drug abuse:* medical registries, the Crime Register, the Suspicion Register, drug-related driving offences, and the Prescribed Drug Register *AUDs:* medical and mortality registries, the Suspicion Register, the Crime Register, and the Prescribed Drug Register	*Drug abuse:* medical registries, the Crime Register, the Suspicion Register, drug-related driving offences, and the Prescribed Drug Register *AUDs:* medical and mortality registries, the Suspicion Register, the Crime Register, and the Prescribed Drug Register	Drug abuse or AUD status of all other relevant biological and step-parents, offspring year of birth, and offspring sex	Yes, drug abuse and AUD registration of not-lived-with biological parents were correlated with offspring drug abuse and AUD (*r* range = 0.14–0.16)	Yes, drug abuse or AUD registration of adoptive or step-parent correlated with offspring drug abuse or AUD (*r* range = 0.04–0.10)	
Kendler et al.^102^	Extended family design	*Snr* 44,250 children of high-risk parents (affected with drug abuse), and offspring of discordant sibling or sibling-in-law Age: 15 and over	*Drug abuse* and *alcohol use disorder:* medical registries, the Crime Register, the Suspicion Register, drug-related driving offences, and the Prescribed Drug Register *Criminal behaviour:* Swedish Crime register *Psychiatric registration:* any mental disorder	*Drug abuse:* medical registers, the Crime Register, the Suspicion Register, drug-related driving offences, and the Prescribed Drug Register	Child sex, year of birth	Not studied	Yes, after accounting for genetic relatedness, parent (and step-parent) drug abuse, AUD, criminal behaviour and psychiatric registration was associated with offspring drug abuse	
Cea & Barnes^108^	Adoption	*VFS* 328 biological and 77 adoption families Age: 14–33 years	*Parenting styles:* offspring report, *family cohesion* (FACES-II), *mother & father care, mother & father overprotectiveness* (PPBI), *parental monitoring, mother and father support, mother and father control* (GBF)	*Polysubstance use:* self-report, composite score, alcohol composition (Volume-Variability Index), smoking, and other drug usage at time 1 (T1: 14–25 years) and T2 (21–33 years)	Age, gender, and adoption status	Not studied	At T1, adoptive family cohesion, parental monitoring, maternal and paternal positive parenting, and father overprotection were associated with offspring substance use (maternal and paternal coercion, maternal overprotectiveness coercion were not). At T2, only cohesion, maternal coercion and overprotection were significant	
Cea & Barnes^104^	Adoption	*VFS* 328 biological and 77 adoption families Age: 14–33 years	*Addiction-prone personality:* self-report, APP-21 *Familial care factor:* mother, father & offspring report, PPBI and FACES-II	*Addiction-prone personality:* self-report, APP-21	Adoption status, and child gender	Not studied	Adoptive parent addiction-prone personality and familial care factor were associated with offspring addiction-prone personality	
Samek et al.^107^	Adoption	*SIBS* 568 adopted and 412 biological offspring Age: 11–25.5 years	*Parental involvement:* offspring report, an average of the maternal and paternal score, PEQ	*Substance use:* self-report, CSUA	Earlier substance use	Not studied	Yes, adoptive parental involvement was negatively associated with offspring substance use	No evidence of passive *rGE* found
Kendler et al.^109^	Sibling comparison	*Snr* 1161 full sibships and 3085 half-sibships of high-risk biological parents; one sibling reared by biological, other by adoptive parents Age: 15 and over	*Adoptive parenting:* protective effect of high-quality rearing environment	*Drug abuse:* medical registers, the Suspicion Register, the Crime Register, drug-related driving offences, and the Prescribed Drug Register	Parental age at birth, high-risk status of the other parent of half-sibling, child gender	Not studied	Children exposed to adoptive parenting had a lower risk of drug abuse than their unexposed siblings, this protective effect disappeared when the adoptive family was disrupted or if there was a high-risk adoptive parent	

*G–E* gene–environment, *G×E* gene–environment interaction, *rGE* gene–environment correlation.

Design = *PGS* Polygenic scores.

Samples = *A-25* Australia 25,000 study, MATCH Mothers and Their Children Study, *MO-MATCH* Missouri Mothers and Their Children Study, *PACER* Parent Alcoholism and Child Environmental Risk Study, *SIBS* Sibling Interaction and Behaviour Study, *Snr* Swedish National Registers*, VET* Vietnam Era Twin Registry, *VFS* Vancouver Family Survey, *V-30* Virginia 30,000 study.

Measures = *APP-21* Addiction-Prone Personality-21 Scale, *CSUA* Computerised Substance Use Assessment, *DIS* Diagnostic Interview Schedule, *DUSI* revised Drug Use Screening Inventory, *FACES-II* Family Adaptability and Cohesion Evaluation Scales II, *GBF* Grace Barnes and Farrell’s 1982 Study*, MAGIC-PC* Missouri Assessment of Genetics Interview for Children–Parent on Child, *PEQ* Parental Environment Questionnaire, *PPBI* Parker Parenting Bonding Instrument, *SAGA* Semi-structured Assessment of the Genetics of Alcoholism.

Statistics = *OR* odds ratio, *HR* hazard ratio, *r* weighted tetrachoric correlation. Effect sizes are not reported for studies that did not investigate both genetic and environmental transmission.

**Table 7:** Detailed characteristics of studies investigating offspring personality (*N* = 6).

Offspring personality								
Study	Design	Sample	Parental attribute (predictor)	Child attribute (outcome)	Control variables	Genetic overlap	Environmental transmission	G–E interplay
Elam et al.^61^	Adoption	*EGDS* 316 families Age: 27 months to 4.5 years	*Adoptive parent hostility:* self-report, IFIRS *Birth mother low behavioural motivation:* self-report, BIBA	*Toddler low social motivation:* observation & parent report, composite score	Prenatal risk and obstetric complications, and adoption openness	Yes, birth mother low behavioural motivation predicted toddler low social motivation (*β* = 0.17)	Yes, adoptive parent hostility predicted offspring disruptive peer behaviour (*β* = 0.11–0.28)	Evocative *rGE*: birth mother low behavioural motivation predicted toddler low social motivation, which predicted adoptive parent–child hostility
Ellingson et al.^71^	Sibling comparison	*CNLSY* 10,251 children of 4,827 mothers Age: 4–14 years	*Smoking during pregnancy:* self-report, mean number of packs smoked per day, reported after pregnancy	*Temperament/personality:* maternal report, CBQ	Maternal age at birth, EA, intelligence, delinquency, offspring sex, birth order, ethnicity, household income, geographic location	Not studied	No difference in temperament/personality between exposed and unexposed siblings	
Van Ryzin et al.^110^	Adoption	*EGDS* 361 families Age: 9 months to 6 years	*Responsive parenting:* observation & self-report, composite score, HOME *Birth parent sociability:* parental self-report, composite score, ATQ	*Social competence:* parent and teacher-report, composite score, SSRS and SCSA	Openness/contact in the adoption, prenatal risk index, child positive emotionality at 9 months	Birth-parent sociability predicted offspring social competence, (*β* = 0.17) but this association did not remain after adjusting for child positive emotionality	Adoptive responsive parenting did not predict offspring social competence	G×E: birth parent sociability x adoptive parent responsive parenting: offspring social competence
Eley et al.^29^	Children-of-twins	*TOSS* 387 MZ, 489 DZ families Age: 11–22	*Neuroticism:* self-report, EPQ	*Neuroticism: s*elf-report, EPQ	Twin sex, and age	No shared genetic effects between parental and offspring neuroticism	Yes, after accounting for genetic relatedness, parental neuroticism was associated with offspring neuroticism (effect size not clear)	
Brooker et al.^111^	Adoption	*EGDS* 505 families Age: 9–18 months	*Child-centred parenting:* observation, three independent coders *Adoptive and birth parent anxiety symptoms:* self-report, BAI	*Social inhibition:* observation, independent coders	Prenatal risk and obstetric complications, adoption openness, adoptive parent EA, and child sex	No, birth parent anxiety did not predict offspring social inhibition	No, adoptive parent–child-centred parenting or anxiety did not predict offspring social inhibition	Evocative *rGE*: birth parent anxiety and child social inhibition predicted adoptive mother–child-centred parenting G×E: birth parent anxiety x adoptive father–child-centred parenting: social inhibition
Kandler et al.^112^	Extended twin	*SPAD* 573 twins and their families Mean age: ~39 years	*Personality dimensions:* self-report, HEXACO, six dimensions: *honesty–humility, emotionality, extraversion, agreeableness, conscientiousness, openness*	*Personality dimensions:* self-report, HEXACO, six dimensions: *honesty–humility, emotionality, extraversion, agreeableness, conscientiousness, openness*	Age, sex	Not studied	No, maternal or paternal shared environment effects were not associated with offspring personality	No evidence of passive *rGE* found

*G–E* gene–environment, *G* *×* *E* gene–environment interaction, *rGE* gene–environment correlation.

Samples = *CNLSY* Children of the National Longitudinal Survey of Youth, *EGDS* Early Growth and Development Study, *SPAD* Study of Personality Architecture and Dynamics*, TOSS* Twin Offspring Study of Sweden.

Measures = *ATQ* Adult Temperament Questionnaire, *BAI* Beck Anxiety Inventory, *BIBA* Behavioural Inhibition/Behavioural Activation scales*, CBQ* Children’s Behaviour Questionnaire, *EPQ* Eysenck Personality Questionnaire, *HEXACO* HEXACO Personality Inventory-Revised, *HOME* Home Observation for Measurement of the Environment, *IFIRS* Iowa Family Interaction Rating Scales, *SSRS* Social Skills Rating System, *SCSA* Social Competence and School Adjustment.

Statistics = *β* standardised parameter estimate. Effect sizes are not reported for studies that did not investigate both genetic and environmental transmission.

### Offspring internalising behaviours

#### Intergenerational transmission of internalising behaviours

Studies investigating the association between parent and offspring internalising behaviours (Table 3), including depression and anxiety, showed substantial evidence of genetic transmission of depressive symptoms^19–22^, and major depressive disorder (MDD) diagnosis^23^. This is in line with twin literature which shows that depression is a heritable phenotype^3^. After accounting for genetic effects, parental depression was associated with offspring internalising behaviours through environmental pathways, and these associations were observed throughout childhood^20,21,24,25^, adolescence^19,26^, and adulthood^23^. Similarly, associations between parental anxiety and offspring internalising behaviours also showed evidence of environmental transmission across development, from toddlerhood to early adulthood^27–32^. However, unlike depression, this association was not partly explained by shared genes, as there was no evidence of genetic overlap between parental anxiety and offspring internalising behaviours^27,29,30,32^. The lack of evidence for genetic transmission of anxiety is at odds with findings from twin literature which estimate that 40% of individual differences in anxiety are explained by genetic factors^3^. However, there are some possible explanations of why genetic transmission is not evident within the adoption and children-of-twins studies reviewed here. Measures of inherited risk in the adoption studies could lack validity, and may not adequately capture the genetic risk of anxiety from birth parents. Alternatively, as longitudinal studies show that genetic factors involved in anxiety change across the lifespan^33^, different genes could be relevant for the occurrence of anxiety in early life and adulthood. Therefore, parental anxiety and offspring internalising symptoms may share fewer common genetic factors that are not easily captured using adoption or children-of-twins designs. Even if different genes are involved in childhood internalising symptoms and adult anxiety, the observed environmental association indicates that exposure to an anxious parent is a risk factor for offspring internalising symptoms.

Overall, environmental associations between parental factors and offspring internalising behaviours were generally driven by exposure to concurrent parental anxiety or depression, whereas prenatal and post-natal symptoms did not have a long-lasting effect^21,24,31,34^. This finding stands in contrast to the substantial body of literature that interprets associations between perinatal maternal distress and offspring mental health outcomes in causal terms^35^. Based on the current findings, such parent–offspring associations detected in previous observational studies are likely to be attributable to unmeasured *rGE*, or concurrent parental depression. In investigating the presence of gene–environment correlation, several adoption studies found no evidence of evocative *rGE*, although some child-to-parent effects were identified^19,28,30,32,36^. These studies highlight the dynamic nature of parent and offspring relationships, where associations can be bidirectional, with both parent and offspring behaviour influencing the other.

#### Parenting behaviours

Children-of-twin studies examining genetic overlap between parenting and offspring mental health found that genes involved in parenting behaviours (such as parental criticism, parental affection and parent–child relationship quality) did not overlap with genes involved in offspring internalising behaviours^37–40^ (Table 3). After accounting for genetic relatedness, several parenting behaviours were associated with offspring internalising behaviours. Negative parenting behaviours, including over-reactive parenting^41^, harsh parenting^36^ and parental criticism^37,40^ were associated with more offspring internalising behaviours, whereas parental expressed affection and a good parent–child relationship quality were associated with positive offspring self-worth^38^, and fewer internalising problems^39^, respectively. Of note, an innovative sibling comparison based on Swedish registry data identified a protective effect of adoptive parenting in children of high-risk biological parents with MDD diagnosis^42^. In interpreting associations between parenting behaviours and offspring outcomes, it is important to again note that these parent–offspring associations can be bidirectional, with each affecting the other over time. Furthermore, parenting behaviours can be evoked by the offspring’s genetically influenced internalising behaviours. However, three adoption studies found no evocative *rGE* effects of offspring internalising symptoms^32,36,37^, although one study reported child-to-parent effects wherein child anger predicted prospective harsh negative parenting^36^.

#### Genetic nurture

Genetic nurture is a relatively new topic within psychiatric genetics, and as such, we identified only two studies that investigated environmentally mediated effects of parental genes on offspring internalising behaviours (Table 3). Both studies were based on the Norwegian Mother, Father and Child (MoBa) sample and estimated variance in offspring depression and anxiety symptoms that was explained by indirect parental genetic effects, over and above the transmission of genes from parent to child. The earlier study, with a smaller sample size, found no evidence for genetic nurture^43^, whereas the subsequent study with three times the sample size identified a genetic nurturing effect on offspring depressive symptoms that was mediated by maternal emotional symptoms^44^. This finding is in line with the studies reviewed above which showed environmental associations between maternal depression and offspring internalising behaviours^20,21,24^ and shows that seemingly environmental associations between parental factors and offspring outcomes may nonetheless be driven by genetically influenced parental traits.

#### Parental educational attainment

A large children-of-twins and siblings study investigating associations between parental educational attainment and offspring depressive symptoms found evidence of genetic, but not environmental transmission^45^ (Table 3). Genetic overlap between education attainment and depression has been reported previously^46^, and this study highlights that without the use of genetically informative designs to account for genetic transmission, phenotypic associations between parental educational attainment and offspring internalising symptoms could be misinterpreted as causal.

#### Parental substance use

A large sibling comparison study investigated associations between maternal alcohol use during pregnancy and offspring emotional problems^47^ (Table 3). Although exposed children were more emotionally reactive and had more somatic complaints than their unexposed siblings, associations between maternal drinking and offspring anxiety and depressive symptoms seemed to be explained by factors shared by siblings born of the same mother. Previous literature investigating the impact of drinking during pregnancy on offspring internalising behaviours shows mixed findings^47^, making it difficult to make firm conclusions on whether there is an environmental association.

### Offspring externalising behaviours

#### Intergenerational transmission of externalising behaviours

Several adoption studies investigating the intergenerational transmission of externalising behaviours (Table 4) were based on the Early Growth and Development Study (EGDS) sample. Detection of effects in these studies seemed to correlate with sample size, indicating that power considerations are important in interpreting these results. In studies with fewer participants (up to 361 families), birth parent externalising behaviour, antisocial behaviour and self-regulation were uncorrelated with offspring externalising behaviours^48–50^, suggesting no shared genetic effects. However, studies with more participants (561 families) showed correlations between birth parent and offspring externalising behaviours^51^, and between birth parent antisocial behaviour and offspring callous–unemotional behaviours^52^, although oppositional and attentional-deficit behaviours were uncorrelated with birth parent antisociality^52^. Findings from previous literature show substantial heritability of externalising behaviours^3^ and highlight the important role of genetic transmission in explaining parent–offspring simarlity^4^. It is likely that the detection of genetic transmission in adoption studies requires more power, especially if the specific parent and offspring phenotypes under investigation are related, but not identical traits.

The role of environmental transmission in externalising behaviours has also been previously implicated^4^. Here, we identified one adoption study which found no robust evidence for an association between parent antisociality and offspring disruptive behaviours^53^. In addition, three large Swedish population-based studies of criminal behaviour found robust evidence of both genetic and environmental transmission of criminal behaviour^8,54,55^ and showed that risk of criminal behaviour was strongest in families where the same parent provided both the genes and the rearing environment^8,55^.

#### Parental anxiety or depression

Evidence from adoption and children-of-twins studies showed genetic overlap between parental depression and offspring externalising behaviours, including ADHD^19–22,56^ (Table 4), whereas associations between overall parental internalising symptoms and offspring externalising symptoms showed mixed results in four adoption studies^25,41,51,57^. Genetic overlap between depression and externalising phenotypes has been reported previously^58^, and the generalist-gene hypothesis suggests that the same genes may pose genetic vulnerabilities toward multiple distinct psychiatric disorders.

After accounting for genetic relatedness, exposure to parental depression was associated with offspring externalising problems in several studies^19–22,24,25,56^, whereas parental anxiety^22^ and overall internalising symptoms^57^ were unrelated to offspring externalising behaviours. Combined with the findings described above, this indicates that exposure to a depressed parent is a risk factor for both internalising and externalising behaviours. As with childhood internalising problems, the association between maternal depression and childhood externalising problems was often observed only in relation to concurrent depressive symptoms^21,22,24^, although one children-of-twins study reported an association between prenatal maternal depression and ADHD in 5-year-olds^56^. Current results mainly highlight that associations with prenatal depression in observational studies that do not control for genetic effects are likely partly explained by unmeasured *rGE*. One adoption study investigating *rGE* reported evocative effects; birth parent depression predicted offspring externalising problems, which in turn predicted adoptive parent depression^19^. As well as demonstrating how genes and environment work in combination, the study highlights the bidirectional relationship between parent and offspring mental health phenotypes.

#### Parenting behaviours

Genetic associations between parenting and offspring externalising behaviours were scarcely investigated (Table 4). A children-of-twins study reported no genetic overlap between parental monitoring and offspring externalising problems^59^, whereas an adoption study reported that birth mother personality characteristics were partially associated with offspring callous–unemotional behaviours^60^. Previous children-of-twins studies show that it is plausible that parents with a predisposition for negative parenting behaviours have offspring predisposed to psychopathology, and subsequently both phenotypes may share a common aetiology^4^.

Studies of environmental transmission found associations between both positive and negative parenting and offspring externalising behaviours. Negative parenting behaviours were associated with increased offspring externalising behaviours^49,53,60–62^, but these effects were sometimes inconsistent across raters. For instance, over-reactive parenting was associated with parent-rated^41,48^, but not teacher-rated^50^ externalising problems. This could reflect differences in the child’s behaviour observed at home by the parent, or at school by the teacher. Alternatively, these differences could be indicative of rater biases resulting from differences in the interpretation of scale items, a unique perception of the children’s behaviour, or the rater’s own mental health^63^. More research is required to clarify rater-specific findings. Focusing on positive parenting, factors such as parental knowledge of offspring whereabouts, good parent–child relationship quality, positive reinforcement, and warm parenting were associated with fewer externalising problems^50–52,59,64^, whereas there were no associations between parental positive reinforcement and ADHD symptoms^52^, or maternal support and offspring delinquent behaviour^65^. Investigation of possible gene-environmental correlation between parenting and offspring externalising behaviours in adoption samples found no passive or evocative *rGE* effects in the associations between parental knowledge and offspring externalising behaviours^59^, whereas one study reported an evocative *rGE* showing that parental hostility was evoked by genetically influenced offspring behaviour^61^, and another reported child-to-parent effects on maternal support and negativity^65^. As well as highlighting the bidirectionality of parent–offspring associations, these studies show that associations between parenting and offspring outcomes vary by phenotype and no single explanation fits all parenting–offspring associations.

#### Parental substance use

Two studies reported that parental drug abuse^66^ and smoking^67^ shared genetic overlap with offspring externalising behaviours (Table 4). These reports of genetic overlap are in line with classical twin studies which suggest that comorbidity between substance use and externalising behaviours is partly due to overlapping genetic factors^68,69^. After accounting for genetic relatedness, mixed evidence for environmental associations between parental substance use and some offspring externalising behaviours was found. Maternal smoking during pregnancy was linked to offspring oppositional defiant disorder^70^ and conduct problems^70^, whereas a larger study showed no association with offspring disruptive behaviours^71^. Similarly, smoking during pregnancy was associated with parent-reported ADHD symptoms in one sibling comparison study^72^, but not another^70^, and was not associated with ADHD diagnosis in a large population-based sample^73^. Exposure to maternal alcohol use during pregnancy was linked to offspring aggression in one study^47^, and to offspring ADHD symptoms in another^74^, but the latter association was not reliably observed across measurement instruments, and moreover, maternal drinking was not associated with ADHD diagnosis^74^ or attentional problems^47^. Studies of parental substance use during childhood found no environmental effect of parent alcohol and tobacco use^64^ or drug abuse^66^ on offspring externalising behaviours. The overall pattern of results indicates that prenatal exposure to substance use may be associated with some offspring externalising behaviours, but no firm conclusions can be drawn from current or previous work.

#### Parental education attainment

Three studies found evidence of genetic overlap between parental education attainment and offspring ADHD symptoms^13,45,75^ (Table 4). Genetic overlap between educational attainment and ADHD is previously known^76^, and is hypothesised to either suggests a common neurobiological process underlying both inattention symptoms and academic achievement, or an indirect mechanism through which genetically influenced inattention impacts academic achievement^77^. Both of these scenarios are feasible in the context of the observed parent–offspring associations.

Findings for an environmental pathway were mixed. Although a within-family PGS study estimated that the association between maternal education and offspring ADHD would be null after adjusting for PGS that captured all heritability based on twin-based estimates^13^, a large children-of-twins study found that maternal education was associated with offspring ADHD symptoms even after accounting for genetic relatedness^45^. Parental educational attainment has been associated with specific parenting styles^78^, and it seems plausible that these parenting behaviours subsequently influence offspring ADHD. However, based on what we know from twin literature, where ADHD shows very high heritability, and little effects of the shared or unique environments^3^, the overall impact of parenting behaviours on ADHD is likely to be small.

#### Genetic nurture

One within-family PGS study of ADHD found no genetic nurturing effect on offspring ADHD due to ADHD or educational attainment related to parental genes^75^. Although this finding requires replication, it is compatible with what we know from twin-based literature, discussed above.

### Offspring educational attainment

#### Intergenerational transmission of educational attainment

Studies investigating intergenerational educational attainment showed consistent evidence of genetic overlap between parent and offspring educational attainment^13,45,79–81^ (Table 5). Additional evidence of genetic transmission was provided by several within-family PGS studies showing that parental genetic liability for educational attainment predicted offspring educational attainment^13,75,82–85^. After accounting for genetic relatedness, evidence of environmental transmission of intergenerational educational attainment was observed in several studies^45,79–82^. Taken together, current literature indicates that as well as passing on education-associated genes, parents may shape the rearing environment in a way that influences the offspring’s subsequent educational attainment. However, these environment influences may nonetheless be partly influenced by parental genes. In line with this, a within-family PGS study provided evidence of passive *rGE*, showing that individuals with higher PGS for educational attainment tended to grow up in better-educated households than those with lower PGS^86^.

#### Genetic nurture

Research into genetic nurture has gained traction in the last two years, starting with the publication of three landmark studies with novel designs to identify genetic nurturing effects on offspring educational attainment^14,83,87^ (Table 5). These studies have highlighted that parental genes can have an indirect (environmentally mediated) effect on offspring educational attainment through parental traits that are genetically influenced. The genetic nurturing effect on offspring educational attainment has been replicated in several samples^15,75,78,84–86,88,89^, and a few studies reported that the observed effect was partly explained by family socioeconomic status^14,15,89^. This finding is compatible with an adoption study which found that adoptive parents with higher income had offspring with increased educational attainment^90^. Other studies reported additional mediating effects of parental IQ^88^, maternal health during pregnancy^89^ and parenting behaviours^78^. The last study was the first to show that specific parenting behaviours are under the genetic influence of education-associated genes, and that these genetically influenced parenting behaviours are subsequently associated with offspring educational attainment. In addition, the study reported evidence of passive *rGE*, as mothers with higher PGS for education attainment provided home environments that were more conducive to higher educational attainment (greater cognitive stimulation, more warm and sensitive parenting, and less chaotic and safer, tidier homes)^78^. Evidence of passive *rGE* was also found for the overall genetic nurturing effect in a within-family PGS study of adoption samples, where parental PGS of educational attainment was more strongly associated with offspring educational attainment in biological families than adoptive families^85^. This particular passive *rGE* has also been reported outside of the reviewed work^91^.

#### Maternal smoking during pregnancy

A large children-of-twins study reported genetic overlap between maternal smoking during pregnancy and offspring general cognitive ability^67^ (Table 5). This finding is in line with the known negative genetic correlation between smoking and educational attainment^92^ and highlights that in observational studies without genetically informative designs, this parent–offspring association explained by unmeasured genetic effects could lead to spurious conclusions. Investigations of environmental transmission did not reveal robust associations; maternal smoking during pregnancy was negatively associated with reading cognition^71^, but associations with other measures of cognitive functioning^71^, general cognitive ability^67^, and academic achievement^67^ did not remain after accounting for genetic relatedness. Previous literature on genetically informative designs suggests that familial factors, including genetic effects, account for the relationship between smoking during pregnancy and offspring cognition^93^.

### Offspring substance use

#### Intergenerational transmission of substance use behaviours

Studies investigating intergenerational transmission of substance use behaviours (Table 6) showed consistent evidence of genetic transmission of substance involvement^94^, alcohol use^9,95–97^, drug abuse^8,9,98,99^ and smoking initiation^100^. There was also evidence of environmental transmission of many substance use behaviours, including drinking behaviour^101^, alcohol use disorder^8,9,95,97^, drug abuse^8,9,11,98,99,102^, smoking behaviour^100,103^ and addiction-prone personalities^104^, whereas parental dependency on alcohol was not consistently associated with offspring alcohol involvement^94,96^. Two studies showed no long-term effects of maternal smoking during pregnancy on offspring substance use behaviours^67,105^. Although parental substance use behaviours were generally associated with an increased likelihood of substance use in offspring, an extended twin study observed negative environmental transmission of smoking behaviours, whereby parental smoking had an inhibiting effect on offspring smoking initiation^103^. The finding was marginally significant and requires replication. One study found evidence of passive *rGE* underlying parent–offspring similarity in drinking behaviours, with more similarities in biological parent–child relationships than in adoptive families^106^.

#### Parenting behaviours

Studies investigating the associations between parenting behaviours and offspring substance use (Table 6) showed that adoptive parenting behaviours such as parental involvement^107^, family care^104^, family cohesion, parental monitoring, parental care and parental support^108^ were associated with a lowered risk of offspring substance use behaviours, whereas adoptive parents’ overprotectiveness or control had no effect^108^. In addition, children exposed to adoptive parenting had a lower risk of drug abuse than their unexposed sibling, indicating a protective effect of adoptive parenting on substance use behaviours, which was also reported for MDD above^109^.

### Offspring personality

There was evidence of genetic and environmental influences underlying associations between parental characteristics and offspring personality (Table 7). Parent sociability and offspring positive emotionality^110^, and parent behavioural motivation and offspring social motivation^61^ shared common genetic factors, whereas the intergenerational transmission of neuroticism seemed to be environmentally explained^29^. There was no evidence of an environmental association between parental traits, including anxiety^111^, sociability^110^, and smoking during pregnancy^71^, and offspring personality traits such as sociability and temperament. In addition, an extended twin study found no evidence of environmental transmission or *rGE* underlying associations between parent and offspring dimensional personality traits^112^. However, two studies observed evocative effects of offspring social behaviours on parenting; adopted offspring’s genetically influenced social behaviours predicted adoptive parent hostility^61^ and child-centred parenting^111^. Overall, current and previous literature indicates that relationships between parental factors and offspring personality vary substantially by phenotype, and can involve both genetic and environmental processes.

## Discussion

This review provides a broad overview of genetically informative literature investigating associations between parental characteristics and offspring mental health and related outcomes. This is a topic of substantial interest, with 89 relevant articles published in the past 6 years. Overall, reviewed studies showed reliable evidence of genetic transmission of depression, criminal behaviour, educational attainment, and substance use behaviours from parent-to-child. Additionally, cross-phenotype genetic overlap was observed in several instances; for example, parental depression, substance use, and educational attainment were all associated with offspring externalising behaviours through genetic pathways (Table 2). After accounting for genetic transmission, parental depression or anxiety were associated with offspring internalising or externalising behaviours through environmental pathways. For maternal exposures, these associations were related to concurrent maternal symptoms, with no long-lasting effect of prenatal depression or anxiety on offspring mental health. Other environmental associations and *rGEs* were observed for parent–offspring similarity in criminal behaviours, substance use behaviours, and educational attainment. In addition, positive and negative parenting behaviours held associations with offspring internalising behaviours, externalising behaviours, substance use behaviours, and educational attainment, with some evidence of *rGE*. Finally, cross-lagged studies showed bidirectional associations between parenting traits and offspring behaviours, where parenting predicted offspring behaviours, and offspring behaviours predicted parenting.

The reviewed literature highlights that genetically informative designs must be implemented to model or control for genetic effects in studies investigating parental influences on offspring development. There was substantial evidence of genetic overlap between parental and offspring phenotypes for both similar traits (e.g. parental depression and offspring internalising symptoms)^19–23^ and dissimilar traits (e.g. parental depression and offspring externalising problems)^19–22,56^. As well as indicating genetic transmission of similar traits, these findings indicate that the same genetic factors may be relevant for the development of several distinct mental health problems^92^, and could also partly explain the comorbidity between mental health disorders that is widely observed in literature^113^. Without accounting for genetic transmission within families, observational studies run a serious risk of misinterpreting these associations as causal environmental influences. For instance, it was observed that after accounting for shared genetic effects, perinatal maternal depression did not hold any long-lasting associations with offspring internalising or externalising behaviours in childhood^21,22,24,31,34^. This is in contrast to the substantial body of literature that interprets associations between perinatal maternal distress and offspring mental health outcomes in causal terms^35^. We urge future studies investigating parent–offspring associations to err on the side of caution in interpreting their results and consider evidence from multiple methodologies in forming their conclusions. Even genetically informative designs can be skewed towards non-genetic findings if there is insufficient power in the study. Triangulating evidence from multiple methodologies is required before a general conclusion can be reached on whether a given parent–offspring association is likely to be truly present, after accounting for shared genetic effects or *rGE*.

Even so, the reviewed studies indicate that both genetic and environmental factors are important in associations between parental factors and offspring mental health outcomes (Table 2). These overall findings raise two important questions; to what extent are parent–offspring associations due to genetic transmission, and to what extent does parenting truly matter? Findings from classical twin literature indicate that between 40 and 80% of individual differences in mental health phenotypes such as internalising and externalising problems between people are explained by additive genetic effects^3^. This suggests that the largest way through which parents influence offspring mental health outcomes is through the passing on of their genes. In addition, estimates of heritability for mental health phenotypes within classical twin literature tend to increase with age, while the influence of the shared family environment decreases^114^. From a developmental perspective, this indicates that genetic influences on offspring mental health become increasingly important as the child gets older while the overall environmental impact of parental characteristics on offspring behaviour is likely to be small. In the current review, effect sizes showing the relative contribution of genetic and environmental factors in parent–offspring associations were not consistently reported and the available statistics are hard to compare between studies. Some studies reported higher effect sizes for genetic or environmental transmission, while others reported equal effect sizes for genetic and environmental effects in parent–offspring associations (Tables 3–7). Based on prior knowledge, the overall effect of any single parental environmental exposure is likely to be far lower than the estimated heritability of offspring mental health and related traits, as is the effect of a single genetic variant. It is also worth highlighting that environmentally mediated influences can still be under the influence of parental genes. Previous twin literature shows that parenting behaviours are under genetic influence themselves and reflect heritable individual differences^115–117^. Genetic nurture is a new way to index the environmentally mediated effect of parental genes on offspring behaviour. The reviewed studies provide evidence of genetic nurture effects on offspring internalising symptoms and educational attainment (Table 2). This is a promising area of research and we expect the development and application of genetic nurture designs to continue to expand in the coming years.

As well as demonstrating genetic overlap and environmental transmission within parent–offspring associations, the reviewed studies showed that confounding by passive *rGE* is also prevalent within genetically informative designs (Table 2). If unmodelled, these unmeasured effects may inflate the estimation of both genetic and environmental factors. Additionally, evocative *rGE* can also explain parent–offspring associations. The reviewed studies showed evidence of evocative *rGEs* underlying associations between parental characteristics and offspring internalising symptoms, externalising symptoms and personality (Table 2). These findings are compatible with previous literature which shows a moderate impact of offspring’s genetically influenced behaviours on parenting factors^118,119^. In instances where evocative *rGE* effects were not observed, child-to-parent effects were sometimes still present^19,28,30,32,36,65^. These findings highlight the bidirectional and dynamic nature of parent–offspring associations, with child-to-parent effects, as well as parent-to-child effects, and also show the importance of cross-lagged models in modelling parent–offspring associations over time.

Reviewed findings with clinical implications are worth highlighting further. Parents with depression, anxiety, substance use problems, and externalising behaviours appeared to pass on these traits to the offspring through both genetic and environmental mechanisms. This information can be used to extend preventative and early intervention services to high-risk children of parents with internalising, externalising, or substance use disorders in healthcare settings. Family-based interventions, including cognitive, behavioural, and psychoeducational components, are already shown to be effective in children of parents with internalising and externalising disorders^120^. In addition, several reviewed studies showed that positive parental environments, such as parental warmth and positive reinforcement, were protective against externalising and substance use behaviours in children with high inherited risk^51,52,109^. Whilst preventative interventions for externalising problems already include a family component, current preventative strategies for substance use incorporate school-based and skills training approaches^121^. A family-based approach could be a valuable addition to preventative interventions of substance use behaviours in early life.

To conclude, parental factors are important predictors of offspring mental health and related outcomes. Both genetic and environmental processes are important in these associations. Further clarification of these processes requires more research. Exciting opportunities for parent–offspring research are increasingly present, with the availability of more datasets and ongoing advances in methodologies.

## References

[1] McLaughlin KA (2012). Parent psychopathology and offspring mental disorders: results from the WHO World Mental Health Surveys. Br. J. Psychiatry.

[2] Martin NG, Eaves LJ (1977). The genetical analysis of covariance structure. Heredity.

[3] Polderman TJC (2015). Meta-analysis of the heritability of human traits based on fifty years of twin studies. Nat. Genet..

[4] McAdams TA (2014). Accounting for genetic and environmental confounds in associations between parent and child characteristics: a systematic review of children-of-twins studies. Psychol. Bull..

[5] Cadoret RJ (1995). Adoption studies. Alcohol Health Res. World.

[6] McAdams TA (2018). Revisiting the children-of-twins design: improving existing models for the exploration of intergenerational associations. Behav. Genet..

[7] Thapar A (2009). Prenatal smoking might not cause attention-deficit/hyperactivity disorder: evidence from a novel design. Biol. Psychiatry.

[8] Kendler KS, Ohlsson H, Sundquist J, Sundquist K, Triparental (2015). Families: a new genetic-epidemiological design applied to drug abuse, alcohol use disorders, and criminal behavior in a swedish national sample. Am. J. Psychiatry.

[9] Kendler KS, Ohlsson H, Sundquist J, Sundquist K (2019). Parent-offspring transmission of drug abuse and alcohol use disorder: application of the multiple parenting relationships design. Am. J. Med. Genet. Part B Neuropsychiatr. Genet..

[10] Lahey, B. B. & D’Onofrio, B. M. All in the family: comparing siblings to test causal hypotheses regarding environmental influences on behavior. *Curr. Directions Psychol. Sci.* **19**, 319–323 (2010). 10.1177/0963721410383977 PMC3643791 23645975

[11] Kendler KS, Ohlsson H, Sundquist J, Sundquist K (2019). A contagion model for within-family transmission of drug abuse. Am. J. Psychiatry.

[12] Wray NR (2014). Research review: polygenic methods and their application to psychiatric traits. J. Child Psychol. Psychiatry.

[13] Pingault, J.-B. et al. Genetic sensitivity analysis: adjusting for genetic confounding in epidemiological associations. Preprint at https://www.biorxiv.org/content/10.1101/592352v2 (2020). 10.1371/journal.pgen.1009590 PMC8238188 34115765

[14] Bates TC (2018). The nature of nurture: using a virtual-parent design to test parenting effects on children’s educational attainment in genotyped families. Twin Res. Hum. Genet..

[15] Bates, T. C. et al. Social competence in parents increases children’s educational attainment: replicable genetically-mediated effects of parenting revealed by non-transmitted DNA. *Twin Res. Human Genet.* **22**, 1–3 (2019). 10.1017/thg.2018.75 30661510

[16] Eaves LJ, Pourcain BS, Smith GD, York TP, Evans DM (2014). Resolving the effects of maternal and offspring genotype on dyadic outcomes in genome wide complex trait analysis (“M-GCTA”). Behav. Genet..

[17] Young AI (2018). Relatedness disequilibrium regression estimates heritability without environmental bias. Nat. Genet..

[18] Eilertsen, E. M. et al. Direct and indirect effects of maternal, paternal, and offspring genotypes: Trio-GCTA. *Behav. Genet*. **51**, 154–161 (2021). 10.1007/s10519-020-10036-6 33387132

[19] McAdams TA (2015). The relationship between parental depressive symptoms and offspring psychopathology: evidence from a children-of-twins study and an adoption study. Psychol. Med..

[20] Grabow AP (2017). Using an adoption-biological family design to examine associations between maternal trauma, maternal depressive symptoms, and child internalizing and externalizing behaviors. Dev. Psychopathol..

[21] Hannigan LJ (2018). Maternal prenatal depressive symptoms and risk for early-life psychopathology in offspring: genetic analyses in the Norwegian Mother and Child Birth Cohort Study. Lancet Psychiatry.

[22] Gjerde, L. C. et al. Associations between maternal depressive symptoms and risk for offspring early-life psychopathology: the role of genetic and non-genetic mechanisms. *Psychol. Med.* 1–9 (2019). 10.1017/S0033291719003301 31813389

[23] Kendler KS, Ohlsson H, Sundquist K, Sundquist J (2018). Sources of parent-offspring resemblance for major depression in a national Swedish extended adoption study. Jama Psychiatry.

[24] Gjerde LC (2017). Maternal perinatal and concurrent depressive symptoms and child behavior problems: a sibling comparison study. J. Child Psychol. Psychiatry.

[25] Hails KA (2019). Interaction between adoptive mothers’ and fathers’ depressive symptoms in risk for children’s emerging problem behavior. Soc. Dev..

[26] Liskola K, Raaska H, Lapinleimu H, Elovainio M (2018). Parental depressive symptoms as a risk factor for child depressive symptoms; testing the social mediators in internationally adopted children. Eur. Child Adolesc. Psychiatry.

[27] Brooker RJ (2014). Birth and adoptive parent anxiety symptoms moderate the link between infant attention control and internalizing problems in toddlerhood. Dev. Psychopathol..

[28] Brooker, R. J. et al. Associations between infant negative affect and parent anxiety symptoms are bidirectional: evidence from mothers and fathers. *Front. Psychol.* **6**, 10.3389/fpsyg.2015.01875 (2015). 10.3389/fpsyg.2015.01875 PMC4667033 26696939

[29] Eley TC (2015). The intergenerational transmission of anxiety: a children-of-twins study. Am. J. Psychiatry.

[30] Ahmadzadeh, Y. I. et al. Anxiety in the family: a genetically informed analysis of transactional associations between mother, father and child anxiety symptoms. *J. Child Psychology Psychiatry Allied Disciplines*. 10.1111/jcpp.13068 (2019). 10.1111/jcpp.13068 PMC6856374 31106427

[31] Gjerde LC (2020). Maternal perinatal and concurrent anxiety and mental health problems in early childhood: a sibling-comparison study. Child Dev..

[32] Field, A. P. et al. Maternal and paternal influences on childhood anxiety symptoms: a genetically sensitive comparison. *J**. Appl. Dev. Psychol.* **68**, 10.1016/j.appdev.2020.101123 (2020). 10.1016/j.appdev.2020.101123 PMC7377633 32704198

[33] Kendler KS, Gardner CO, Lichtenstein P (2008). A developmental twin study of symptoms of anxiety and depression: evidence for genetic innovation and attenuation. Psychol. Med..

[34] Bekkhus M (2018). Re-examining the link between prenatal maternal anxiety and child emotional difficulties, using a sibling design. Int. J. Epidemiol..

[35] Glover V, O’Connor TG (2002). Effects of antenatal stress and anxiety: implications for development and psychiatry. Br. J. Psychiatry.

[36] Bridgett, D. J. et al. Contributions of mothers’ and fathers’ parenting to children’s self-regulation: evidence from an adoption study. *Dev. Sci.* **21**, 10.1111/desc.12692 (2018). 10.1111/desc.12692 PMC6202135 29978935

[37] Horwitz BN (2015). Parental criticism is an environmental influence on adolescent somatic symptoms. J. Fam. Psychol..

[38] McAdams TA (2017). Associations between the parent-child relationship and adolescent self-worth: a genetically informed study of twin parents and their adolescent children. J. Child Psychol. Psychiatry.

[39] Hannigan LJ (2018). Shared genetic influences do not explain the association between parent-offspring relationship quality and offspring internalizing problems: results from a Children-of-Twins study. Psychol. Med..

[40] Ahmadzadeh, Y. I. et al. Parental criticism and adolescent internalising symptoms: associations remain after accounting for shared genetic effects. Preprint at *medRxiv*10.1101/2020.05.07.20084319 (2020).

[41] Marceau K (2015). Combined influences of genes, prenatal environment, cortisol, and parenting on the development of children’s internalizing versus externalizing problems. Behav. Genet..

[42] Kendler KS, Ohlsson H, Sundquist J, Sundquist K (2020). The rearing environment and risk for major depression: a Swedish national high-risk home-reared and adopted-away co-sibling control study. Am. J. Psychiatry.

[43] Jami ES (2020). Maternal and paternal effects on offspring internalizing problems: results from genetic and family-based analyses. Am. J. Med. Genet. Part B Neuropsychiatr. Genet..

[44] Cheesman R (2020). How important are parents in the development of child anxiety and depression? A genomic analysis of parent-offspring trios in the Norwegian Mother Father and Child Cohort Study (MoBa). BMC Med..

[45] Torvik, F. A. et al. Mechanisms linking parental educational attainment with child ADHD, depression, and academic problems: a study of extended families in The Norwegian Mother, Father and Child Cohort Study. *J. Child Psychol. Psychiatry* **61**, 1009–1018 (2020). 10.1111/jcpp.13197 PMC8607471 31957030

[46] Jansen PR (2018). Polygenic scores for schizophrenia and educational attainment are associated with behavioural problems in early childhood in the general population. J. Child Psychol. Psychiatry.

[47] Lund IO (2019). Is the association between maternal alcohol consumption in pregnancy and pre‐school child behavioural and emotional problems causal? Multiple approaches for controlling unmeasured confounding. Addiction.

[48] Lipscomb ST (2014). Genetic vulnerability interacts with parenting and early care and education to predict increasing externalizing behavior. Int. J. Behav. Dev..

[49] Stover CS (2016). Marital hostility, hostile parenting, and child aggression: associations from toddlerhood to school age. J. Am. Acad. Child Adolesc. Psychiatry.

[50] Reuben JD (2016). Warm parenting and effortful control in toddlerhood: independent and interactive predictors of school-age externalizing behavior. J. Abnorm. Child Psychol..

[51] Marceau K (2019). Parenting and prenatal risk as moderators of genetic influences on conduct problems during middle childhood. Dev. Psychol..

[52] Hyde LW (2016). Heritable and nonheritable pathways to early callous-unemotional behaviors. Am. J. Psychiatry.

[53] Bornovalova MA (2014). Understanding the relative contributions of direct environmental effects and passive genotype-environment correlations in the association between familial risk factors and child disruptive behavior disorders. Psychol. Med..

[54] Kendler KS (2014). A Swedish national adoption study of criminality. Psychol. Med..

[55] Kendler KS, Ohlsson H, Morris NA, Sundquist J, Sundquist K (2015). A Swedish population-based study of the mechanisms of parent-offspring transmission of criminal behavior. Psychol. Med..

[56] Eilertsen, E. M. et al. Parental prenatal symptoms of depression and offspring symptoms of ADHD: a genetically informed intergenerational study. *J. Attention Disord.*10.1177/1087054720914386 (2020). 10.1177/1087054720914386 32338109

[57] Roos LE (2016). Inherited and environmental influences on a childhood co-occurring symptom phenotype: evidence from an adoption study. Dev. Psychopathol..

[58] Howard DM (2019). Genome-wide meta-analysis of depression identifies 102 independent variants and highlights the importance of the prefrontal brain regions. Nat. Neurosci..

[59] Marceau K (2015). Parental knowledge is an environmental influence on adolescent externalizing. J. Child Psychol. Psychiatry.

[60] Trentacosta CJ (2019). Callous-unemotional behaviors and harsh parenting: reciprocal associations across early childhood and moderation by inherited risk. J. Abnorm. Child Psychol..

[61] Elam KK (2014). Adoptive parent hostility and children’s peer behavior problems: examining the role of genetically informed child attributes on adoptive parent behavior. Dev. Psychol..

[62] Plamondon A, Browne DT, Madigan S, Jenkins JM (2018). Disentangling child-specific and family-wide processes underlying negative mother-child transactions. J. Abnorm. Child Psychol..

[63] Hoyt WT (2000). Rater bias in psychological research: when is it a problem and what can we do about it?. Psychol. methods.

[64] Samek DR (2014). General and specific predictors of nicotine and alcohol dependence in early adulthood: genetic and environmental influences. J. Stud. Alcohol Drugs.

[65] Guimond FA (2016). Associations between mother-child relationship quality and adolescent adjustment: using a genetically controlled design to determine the direction and magnitude of effects. Int. J. Behav. Dev..

[66] Kendler KS, Ohlsson H, Sundquist K, Sundquist J (2016). Cross-generational transmission from drug abuse in parents to attention-deficit/hyperactivity disorder in children. Psychol. Med..

[67] Kuja-Halkola R, D’Onofrio BM, Larsson H, Lichtenstein P (2014). Maternal smoking during pregnancy and adverse outcomes in offspring: genetic and environmental sources of covariance. Behav. Genet..

[68] Hicks BM, Iacono WG, McGue M (2012). Index of the transmissible common liability to addiction: heritability and prospective associations with substance abuse and related outcomes. Drug alcohol Depend..

[69] Kendler KS, Myers J (2014). The boundaries of the internalizing and externalizing genetic spectra in men and women. Psychol. Med.

[70] Estabrook R (2016). Separating family-level and direct exposure effects of smoking during pregnancy on offspring externalizing symptoms: bridging the behavior genetic and behavior teratologic divide. Behav. Genet..

[71] Ellingson, J. M., Goodnight, J. A., Van Hulle, C. A., Waldman, I. D. & D’Onofrio, B. M. A sibling-comparison study of smoking during pregnancy and childhood psychological traits. *Behav. Genet.* **44**, 25–35 (2014). 10.1007/s10519-013-9618-6 PMC3947202 24085497

[72] Knopik VS (2016). Smoking during pregnancy and ADHD risk: a genetically informed, multiple-rater approach. Am. J. Med. Genet. Part B Neuropsychiatr. Genet..

[73] Obel, C. et al. The risk of attention deficit hyperactivity disorder in children exposed to maternal smoking during pregnancy—a re-examination using a sibling design. *J. Child Psychol. Psyciatry* **57**, 532–537 (2016). 10.1111/jcpp.12478 26511313

[74] Eilertsen EM (2017). Maternal alcohol use during pregnancy and offspring attention-deficit hyperactivity disorder (ADHD): a prospective sibling control study. Int. J. Epidemiol..

[75] de Zeeuw, E. L. et al. Intergenerational transmission of education and ADHD: effects of parental genotypes. *Behav. Genet*. **50**, 221–232 (2020). 10.1007/s10519-020-09992-w PMC7355279 32026073

[76] Demontis D (2019). Discovery of the first genome-wide significant risk loci for attention deficit/hyperactivity disorder. Nat. Genet..

[77] Liu C-Y, Li Y, Viding E, Asherson P, Pingault J-B (2019). The developmental course of inattention symptoms predicts academic achievement due to shared genetic aetiology: a longitudinal twin study. Eur. Child Adolesc. Psychiatry.

[78] Wertz, J. et al. Using DNA from mothers and children to study parental investment in children’s educational attainment. *Child Dev.*10.1111/cdev.13329 (2019). 10.1111/cdev.13329 PMC7183873 31657015

[79] Ayorech Z, Krapohl E, Plomin R, von Stumm S (2017). Genetic influence on intergenerational educational attainment. Psychol. Sci..

[80] Borriello, G. A. et al. The intergenerational transmission of mathematics achievement in middle childhood: a prospective adoption design. *Dev. Sci.*10.1111/desc.12974 (2020). 10.1111/desc.12974 PMC7581538 32324330

[81] Halpern-Manners, A. et al. The intergenerational transmission of early educational advantages: new results based on an adoption design. *Res. Soc. Stratif. Mobil.* **67**, 10.1016/j.rssm.2020.100486 (2020). 10.1016/j.rssm.2020.100486 PMC7386403 32724268

[82] Conley D (2015). Is the effect of parental education on offspring biased or moderated by genotype?. J. Soc. Sci..

[83] Kong A (2018). The nature of nurture: effects of parental genotypes. Science.

[84] Liu HX (2018). Social and genetic pathways in multigenerational transmission of educational attainment. Am. Soc. Rev..

[85] Domingue, B. W. & Fletcher, J. Separating measured genetic and environmental effects: evidence linking parental genotype and adopted child outcomes. *Behav. Genet.*10.1007/s10519-020-10000-4 (2020). 10.1007/s10519-020-10000-4 PMC7442617 32350631

[86] Belsky DW (2018). Genetic analysis of social-class mobility in five longitudinal studies. Proc. Natl Acad. Sci. USA.

[87] Young AI, Benonisdottir S, Przeworski M, Kong A (2019). Deconstructing the sources of genotype-phenotype associations in humans. Science.

[88] Willoughby, E. A., McGue, M., Iacono, W. G., Rustichini, A. & Lee, J. J. The role of parental genotype in predicting offspring years of education: evidence for genetic nurture. *Mol. Psychiatry* 1–9 (2019). 10.1038/s41380-019-0494-1 PMC7061492 31444472

[89] Armstrong-Carter, E. et al. The earliest origins of genetic nurture: the prenatal environment mediates the association between maternal genetics and child development. *Psychol. Sci.*10.1177/0956797620917209 (2020). 10.1177/0956797620917209 PMC7370247 32484377

[90] Scheeren L, Das M, Liefbroer AC (2017). Intergenerational transmission of educational attainment in adoptive families in the Netherlands. Res. Soc. Stratif. Mobil..

[91] Cheesman R (2020). Comparison of adopted and nonadopted individuals reveals gene-environment interplay for education in the UK Biobank. Psychol. Sci..

[92] Bulik-Sullivan B (2015). An atlas of genetic correlations across human diseases and traits. Nat. Genet..

[93] D’Onofrio BM, Lahey BB, Turkheimer E, Lichtenstein P (2013). Critical need for family-based, quasi-experimental designs in integrating genetic and social science research. Am. J. Public Health.

[94] Waldron M (2014). Parental separation and early substance involvement: results from children of alcoholic and cannabis dependent twins. Drug Alcohol Depend..

[95] Kendler KS (2015). An extended Swedish National Adoption Study Of Alcohol Use Disorder. JAMA Psychiatry.

[96] Grant JD (2015). Parental separation and offspring alcohol involvement: findings from offspring of alcoholic and drug dependent twin fathers. Alcohol Clin. Exp. Res..

[97] Kendler KS, Ohlsson H, Edwards A, Sundquist J, Sundquist K (2017). The clinical features of alcohol use disorders in biological and step-fathers that predict risk for alcohol use disorders in offspring. Am. J. Med. Genet. Part B Neuropsychiatr. Genet..

[98] Kendler KS, Ohlsson H, Sundquist K, Sundquist J (2015). The causes of parent-offspring transmission of drug abuse: a Swedish population-based study. Psychol. Med..

[99] Kendler KS, Ohlsson H, Sundquist K, Sundquist J (2018). Sources of parent-child transmission of drug abuse: path analyses of not-lived-with parental, stepparental, triparental, and adoptive families. J. Nerv. Ment. Dis..

[100] Treur JL (2018). Testing familial transmission of smoking with two different research designs. Nicotine Tob. Res..

[101] McGue M (2007). The environments of adopted and non-adopted youth: evidence on range restriction from the Sibling Interaction and Behavior Study (SIBS). Behav. Genet..

[102] Kendler KS, Ohlsson H, Sundquist J, Sundquist K (2020). Facilitating versus inhibiting the transmission of drug abuse from high-risk parents to their children: a Swedish National Study. Twin Res. Hum. Genet..

[103] Maes HH (2018). Cross-cultural comparison of genetic and cultural transmission of smoking initiation using an extended twin kinship model. Twin Res. Hum. Genet..

[104] Cea NF, Barnes GE (2015). The development of addiction-prone personality traits in biological and adoptive families. Personal. Individ. Differences.

[105] Bidwell LC (2017). Prenatal exposure effects on early adolescent substance use: preliminary evidence from a genetically informed ayesian approach. J. Stud. Alcohol Drugs.

[106] McGue M, Malone S, Keyes M, Iacono WG (2014). Parent-offspring similarity for drinking: a longitudinal adoption study. Behav. Genet..

[107] Samek DR, Rueter MA, Keyes MA, McGue M, Iacono WG (2015). Parent involvement, sibling companionship, and adolescent substance use: a longitudinal, genetically informed design. J. Fam. Psychol..

[108] Cea NF, Barnes GE (2014). Parenting styles and offspring’s polysubstance use in biological and adoptive families. Int. J. Child Youth Fam. Stud..

[109] Kendler KS, Ohlsson H, Sundquist K, Sundquist J (2016). The rearing environment and risk for drug abuse: a Swedish national high-risk adopted and not adopted co-sibling control study. Psychol. Med..

[110] Van Ryzin MJ (2015). Genetic influences can protect against unresponsive parenting in the prediction of child social competence. Child Dev..

[111] Brooker RJ (2016). Early inherited risk for anxiety moderates the association between fathers’ child-centered parenting and early social inhibition. J. Dev. Orig. Health Dis..

[112] Kandler C, Richter J, Zapko-Willmes A (2019). The nature and nurture of HEXACO personality trait differences an extended twin family study. Z. Fur Psychologie J. Psychol..

[113] Lai HMX, Cleary M, Sitharthan T, Hunt GE (2015). Prevalence of comorbid substance use, anxiety and mood disorders in epidemiological surveys, 1990–2014: a systematic review and meta-analysis. Drug Alcohol Depend..

[114] Bergen SE, Gardner CO, Kendler KS (2007). Age-related changes in heritability of behavioral phenotypes over adolescence and young adulthood: a meta-analysis. Twin Res. Hum. Genet..

[115] Plomin R, Reiss D, Hetherington EM, Howe GW (1994). Nature and nurture: genetic contributions to measures of the family environment. Dev. Psychol..

[116] Klahr AM, Burt SA (2014). Elucidating the etiology of individual differences in parenting: a meta-analysis of behavioral genetic research. Psychol. Bull..

[117] Vinkhuyzen AAE, Van Der Sluis S, De Geus EJC, Boomsma DI, Posthuma D (2010). Genetic influences on ‘environmental’ factors. Genes, Brain Behav..

[118] Avinun R, Knafo A (2013). Parenting as a reaction evoked by children’s genotype: a meta-analysis of children-as-twins studies. Personal. Soc. Psychol. Rev..

[119] Kendler KS, Baker JH (2007). Genetic influences on measures of the environment: a systematic review. Psychol. Med..

[120] Siegenthaler E, Munder T, Egger M (2012). Effect of preventive interventions in mentally ill parents on the mental health of the offspring: systematic review and meta-analysis. J. Am. Acad. Child Adolesc. Psychiatry.

[121] Stockings E (2016). Prevention, early intervention, harm reduction, and treatment of substance use in young people. Lancet Psychiatry.

[122] Keller MC (2009). Modeling extended twin family data I: description of the Cascade model. Twin Res Hum. Genet..

[123] O’Reilly LM (2020). The intergenerational transmission of suicidal behavior: an offspring of siblings study. Transl. Psychiatry.

[124] Kendler KS, Turkheimer E, Ohlsson H, Sundquist J, Sundquist K (2015). Family environment and the malleability of cognitive ability: a Swedish national home-reared and adopted-away cosibling control study. Proc. Natl Acad. Sci. USA.

